# EEG and MEG Data Analysis in SPM8

**DOI:** 10.1155/2011/852961

**Published:** 2011-03-06

**Authors:** Vladimir Litvak, Jérémie Mattout, Stefan Kiebel, Christophe Phillips, Richard Henson, James Kilner, Gareth Barnes, Robert Oostenveld, Jean Daunizeau, Guillaume Flandin, Will Penny, Karl Friston

**Affiliations:** ^1^The Wellcome Trust Centre for Neuroimaging, UCL Institute of Neurology, Queen Square, London WC1N 3BG, UK; ^2^INSERM U1028, CNRS UMR5292, Lyon Neuroscience Research Centre, Brain Dynamics and Cognition Team, Lyon, F-69500, France; ^3^Max Planck Institute for Human Cognitive and Brain Sciences, 04303 Leipzig, Germany; ^4^Cyclotron Research Centre, University of Liège, 4000 Liège, Belgium; ^5^MRC Cognition and Brain Sciences Unit, Cambridge CB2 7EF, UK; ^6^Donders Institute for Brain, Cognition, and Behaviour, Radboud University Nijmegen, 6500 HB Nijmegen, The Netherlands

## Abstract

SPM is a free and open source software written in MATLAB (The MathWorks, Inc.). In addition to standard M/EEG preprocessing, we presently offer three main analysis tools: (i) statistical analysis of scalp-maps, time-frequency images, and volumetric 3D source reconstruction images based on the general linear model, with correction for multiple comparisons using random field theory; (ii) Bayesian M/EEG source reconstruction, including support for group studies, simultaneous EEG and MEG, and fMRI priors; (iii) dynamic causal modelling (DCM), an approach combining neural modelling with data analysis for which there are several variants dealing with evoked responses, steady state responses (power spectra and cross-spectra), induced responses, and phase coupling. SPM8 is integrated with the FieldTrip toolbox , making it possible for users to combine a variety of standard analysis methods with new schemes implemented in SPM and build custom analysis tools using powerful graphical user interface (GUI) and batching tools.

## 1. Introduction

Statistical parametric mapping (SPM) is a free and open source academic software distributed under GNU General Public License. The aim of SPM is to communicate and disseminate methods for neuroimaging data analysis to the scientific community that have been developed by the SPM coauthors associated with the Wellcome Trust Centre for Neuroimaging, UCL Institute of Neurology.

The origins of SPM software go back to 1990, when SPM was first formulated for the statistical analysis of positron emission tomography (PET) data [1, 2]. The software incorporated several important theoretical advances, such as the use of general linear model (GLM) to describe, in a generic way, a variety of experimental designs [3] and random field theory (RFT) to solve the problem of multiple comparisons arising from the application of mass univariate tests to images with multiple voxels [4]. As functional magnetic resonance imaging (fMRI) gained popularity later in the decade, SPM was further developed to support this new imaging modality, introducing the notion of a hemodynamic response function and associated convolution models for serially correlated time series. This formulation became an established standard in the field and most other free and commercial packages for fMRI analysis implement variants of it. In parallel, increasingly more sophisticated tools for registration, spatial normalization, and segmentation of functional and structural images were developed [5]. In addition to finessing fMRI and PET analyses, these methods made it possible to apply SPM to structural MRIs [6], which became the field of voxel-based morphometry (VBM). 

The first decade of the 21st century brought about two further key theoretical developments for SPM: increasing use of Bayesian methods (e.g., posterior probability mapping [7]) and a focus on methods for studying functional integration rather than specialization. Dynamic causal modelling (DCM [8]) was introduced as a generic method for studying functional integration in neural systems. This approach uses Bayesian methods for fitting dynamic models (formulated as systems of differential equations) to functional imaging data, making inferences about model parameters and performing model comparison. Bayesian model comparison uses an approximation to the model evidence (probability of the data given the model). Model evidence quantifies the properties of a good model; that is, that it explains the data as accurately as possible and, at the same time, has minimal complexity [9–11]. Further development and refinement of DCM and related methods are likely to remain the focus of research in the future.

In the second half of the decade, the research focus of the SPM group shifted towards the analysis of MEG and EEG (M/EEG). This resulted in three main developments. First, the “classical” SPM approach was extended to the analysis of M/EEG scalp maps [12–14] and time-frequency images [15]. Second, a new approach to electromagnetic source reconstruction was introduced based on Bayesian inversion of hierarchical Gaussian process models [16–18]. The Bayesian perspective was also applied to the problem of equivalent current dipole modelling [19]. Third, DCM was extended to M/EEG data and several variants of the approach were validated, focusing on evoked responses [20], induced responses [21], steady state responses [22], and phase coupling [23]. In order to make it possible for our colleagues to apply these methods easily to their data, infrastructure for conversion and pre-processing of M/EEG data from a wide range of recording systems were incorporated in the SPM software, with notable contribution from the developers of the FieldTrip software (http://www.ru.nl/donders/fieldtrip, see Oostenveld et al. in this issue). SPM for M/EEG is constructed to support the high-level functionality developed by our group and is not intended as a generic repository of useful methods. This distinguishes SPM from some other toolboxes (e.g., FieldTrip).

The present paper focuses on the implementation of these tools in the most recent SPM version, SPM8. We will not rehearse all the technical details of the methods, for which the reader is referred to the relevant papers. Moreover, we will also avoid focusing on specific interface details, as these often change with intensive SPM development. The details we do mention are correct for SPM8 version 4010, released on July 21, 2010. Our aim is to provide an overview of SPM functionality and the data analysis pathways that the software presently supports. This overview is quite long and inclusive. This reflects the fact that the software covers three distinct domains, source reconstruction, statistical parametric mapping (topological inference over various spaces) and dynamic causal modeling. Each entails a set of assumptions and procedures, some of which are fairly basic and common to most analyses of electromagnetic data and some of which are unique to the applications we consider. We have elected to cover all the basic issues for completeness and to relate them to the specific issues within each domain. However, the readers who are familiar with the basic parts could easily skip these sections. The paper is organized as follows. After presenting a brief overview of the SPM8 user interface, we focus on each of the three core parts of SPM for M/EEG presented above: (i) statistical analysis of images, (ii) Bayesian source reconstruction, and (iii) DCM for M/EEG. The Appendix describes the M/EEG pre-processing infrastructure in SPM8 and explains how to get from raw M/EEG data to the format suitable for analysis with one of the core SPM methods.

## 2. SPM8 Interface and Overview

The SPM software consists of a library of MATLAB M-files and a small number of C-files, using the MATLAB MEX gateway for the most computer-intensive operations. Its installation simply consists of unpacking a ZIP archive on the user computer and adding the root SPM directory to the MATLAB path. More details on the installation (especially compilation of the MEX files if needed) can be found on the SPM wiki on Wikibooks (http://en.wikibooks.org/wiki/SPM). SPM requires a prior installation of MATLAB, a commercial high-end numerical software platform developed by the MathWorks, Inc. (Natick, USA). More specifically, SPM requires version R14SP3 (released in 2005) or any more recent version (up to the latest R2010b). It runs on any platform supported by MATLAB, that is, Microsoft Windows, Macintosh OS, and Linux, 32- and 64-bit. A standalone version of SPM8, compiled using the MATLAB compiler, is available upon request—it allows using most of the SPM functionalities without requiring the availability of a MATLAB licence. 

**Figure 1:**
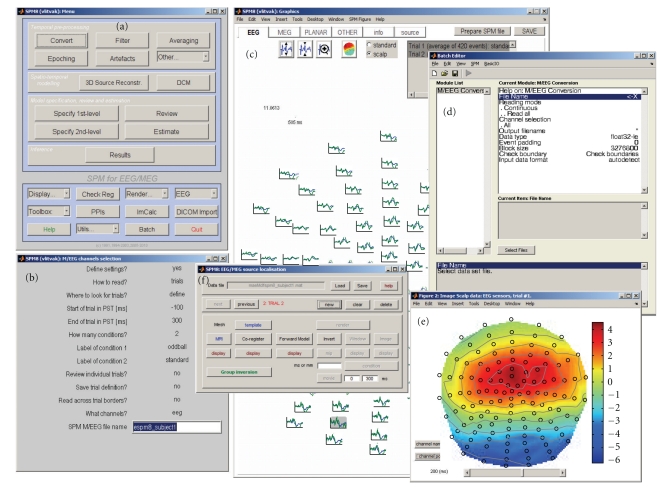
SPM8 for M/EEG graphical user interface tools; see also Figure 11. (a) Menu window, (b) interactive window showing a series of inputs required for conversion of an EEG dataset, (c) graphics window with the SPM8 for M/EEG reviewing tool. Evoked responses recorded in a mismatch negativity experiment are displayed in a topographical plot, (d) MATLAB batch tool with the configuration interface for data conversion, (e) Scalp map of potential distribution for mismatch negativity data that was created from the reviewing tool, and (f) 3D source reconstruction interface.

SPM for M/EEG can be invoked by typing **spm eeg** on the MATLAB command line and pressing Enter. After a brief initialization, the SPM GUI will appear. It consists of three windows (see Figure 1). The menu window on the top left (Figure 1(a)) contains buttons and other GUI elements used to access different SPM functions. This window's contents only change if the user switches modalities (fMRI/PET/MEEG). The interactive window (bottom left, Figure 1(b)) is used by SPM functions for creating dynamic GUI elements, when necessary (for instance, to present the user with a choice or ask for input). The graphics window on the right (Figure 1(c)) is where SPM presents intermediate and final results of its analyses. It is also used by the SPM M/EEG reviewing tool. Additional graphics windows are created when necessary. 

There are three ways to access SPM M/EEG functionality. The first is to use the GUI. Since SPM8 is a GUI-based application, all the standard pre-processing and analysis procedures can be accessed this way with no need for programming. We recommend that beginners use the GUI first, because this will prompt SPM to ask for all relevant information needed to process the data. The second way is to use the matlabbatch tool (Figure 1(d)). Matlabbatch (http://sourceforge.net/projects/matlabbatch/) is a standalone batch system for MATLAB developed by Volkmar Glauche, based on the job manager, originally developed for SPM5. Matlabbatch basically allows “programming without programming”. Processing pipelines can be built and configured using a specialized batch GUI and then applied to multiple datasets in noninteractive mode. The batch system is designed for repetitive analyses of data, once the user knows what should be done, and in which order. Matlabbatch can be accessed by pressing the “Batch” button in the SPM menu window. This will open the batch tool window. SPM functionality can be accessed via the “SPM” menu in this window. Finally, users familiar with MATLAB programming can call SPM functions directly from their scripts without using the GUI. We will refer to this way of using SPM as “scripting” as opposed to “batching”; that is, using the batch tool. The use of GUI, batching, and scripting are not always clearly separated, as for some functions the batch tool is the only available GUI. Also, batch pipelines can be created, modified, and run via scripts. In fact, creating a template batch and then invoking it from a script with specific inputs is the most convenient way to prescribe some of the more complicated analyses in SPM. Thus, SPM scripts can combine the user's own code with invoking SPM functions directly or via batch pipelines. The facilities used by SPM programmers to create dynamic GUIs and batch tools are also available to users for their own custom tools. 

All the analysis procedures in SPM are optimized to reduce computation time: typically an analysis of a single dataset (e.g., source reconstruction or DCM) can be completed in minutes (or in the worst case, tens of minutes) on a standard desktop computer. SPM does not require any special computer infrastructure or parallel computations, although one of our development directions is to introduce parallelization to finesse analysis of multiple subjects and fitting multiple alternative models to the data.

In what follows, we consider the three main domains in which SPM functionality is used. We start with analyses of M/EEG data in sensor space and then proceed to source space analyses in the subsequent sections. Typically, sensor-level analyses are used to identify peristimulus time or frequency windows, which are the focus of subsequent analyses in source space. These sensor space analyses use, effectively, standard SPM procedures (topological inference) applied to a variety of electromagnetic data features that are organised into images.

## 3. Sensor-Level Analysis and Topological Inference

EEG and MEG typically produce a time-varying modulation of signal amplitude or frequency-specific power in some peristimulus time period, at each electrode or sensor. Often, researchers are interested in whether condition-specific effects (observed at particular sensors and peristimulus times) are statistically significant. However, this inference must correct for the number of statistical tests performed. One way to do so is to control the family-wise error rate (FWER), the probability of making a false positive over the whole search space [24]. For independent observations, the FWER scales with the number of observations. A simple method for controlling FWER is the Bonferroni correction. However, this procedure is rarely adopted in neuroimaging because it assumes that neighbouring observations are independent. When there is a high degree of correlation among neighbouring samples (e.g., when data features are smooth), this correction is far too conservative.

Although the multiple comparisons problem has always existed for M/EEG analyses (due to the number of time bins in the peristimulus time window), the need for a correction method has become more acute with the advent of high-density EEG caps and MEG sensor arrays that increase the number of observations across the scalp. In many analyses, the multiple comparisons problem is circumvented by restricting the search space prior to inference, so that there is only one test per repeated measure. This is usually accomplished by averaging the data over prespecified sensors and time bins of interest. This produces one summary statistic per subject per condition. In many instances, this is a powerful and valid way to sidestep the multiple comparisons problem; however, it requires the space of interest to be specified *a priori*. A principled specification of this space could use orthogonal or independent data features. For example, if one were interested in the attentional modulation of the N170 (a typical event-related wave recorded 170 ms after face presentation), one could first define the electrodes and time bins that expressed an N170 (compared to baseline) and then test for the effects of attention on their average. Note that this approach assumes that condition-specific effects occur at the same sensors and time, and is only valid when selection is not biased [25]. In situations where the location of evoked or induced responses is not known *a priori* or cannot be localized independently, one can use topological inference to search over some space for significant responses; this is the approach implemented in SPM. It is based on the random field theory (RFT [4]). RFT provides a way of adjusting the *P*-values that takes into account the fact that neighbouring sensors are not independent, by virtue of continuity in the original data. Provided the data are smooth, the RFT adjustment is less severe (i.e., is more sensitive) than a Bonferroni correction for the number of sensors. The theoretical basis of topological inference for M/EEG has been recently reviewed by Kilner and Friston [14]. Here, we rehearse some of the points from this review and provide more details about the SPM implementation of the method.

Statistical analyses of M/EEG data in SPM use the same mechanisms as all other data types (PET, fMRI, and structural MRI in VBM). This simply requires transforming data from SPM M/EEG format to image files (NIfTI format, http://nifti.nimh.nih.gov/nifti-1/). Once the data are in this image format, statistical analyses for M/EEG are procedurally identical to between-subject analyses of PET or VBM data (e.g., second level analyses in fMRI [26]). These analyses assume one summary statistic image per subject per condition (or level of an experimental factor). Here, a summary statistic image is just a technical term for the data feature summarising treatment effects that one wants to make an inference about. More formally, when this summary statistic is itself a maximum likelihood estimate based on within-subject data, the analysis is called a summary-statistic procedure for random effect models. In the present context, we will see that the summary statistic can comprise many different data features.

### 3.1. Creating Summary Statistics: Conversion to Images

This function takes SPM M/EEG sensor data as input and generates an image for each trial (for trials that were not rejected). This analysis can be applied to EEG and MEG (separately). In the case of MEG systems with planar gradiometers, images can be generated from root-mean-square values combining the two planar gradiometers at each location. In an averaged dataset, this will produce a single image per condition and enable statistical comparisons across subjects. In an epoched dataset, there will be an image per trial and multiple images per condition. It is, therefore, possible to perform within-subject statistical tests and then also take further summary statistic images (usually contrasts of parameter estimates from the within-subject models) from each subject to second level analyses between subjects.

#### 3.1.1. Images over Time

**Figure 2:**
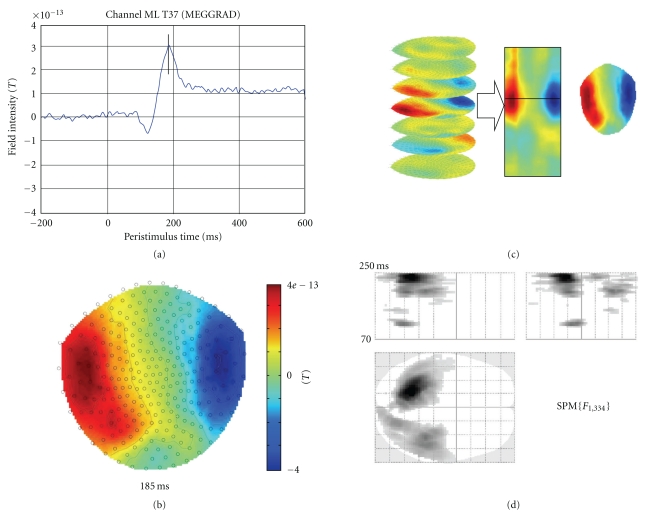
Construction of (space × space × time) summary statistic image and the ensuing SPM inference. The data are MEG responses to presentation of images of faces and scrambled faces. (a) Average ERF for a single subject recorded at a left temporal sensor in response to face presentation. The vertical line indicates the maximum positive value of this ERF. (b) Sensor-space map interpolated across all sensors at 185 ms after the stimulus, indicated by the line in (a). (c) Construction of a 3D (space × space × time) data volume from sensor-space maps, such as shown in (b). (d) Results of F test for difference between responses to faces and scrambled faces. Overall, single trials (168 for each condition) were converted to images as shown in (c). A two-sample *t*-test was performed and the results were assessed with an *F*-contrast to test for differences of either polarity. The results were thresholded at *P* = .05 with FWER correction based on random field theory. The red arrow indicates the peak value of the *F*-statistic (at 245 ms).

Data in the time domain are converted into an image by generating a scalp map for each time frame and stacking scalp maps over peristimulus time (see Figure 2). Scalp maps are generated using the 2D sensor layout specified in the dataset (see Appendix B.3) and linear interpolation between sensors. The user is asked to specify the output dimensions of the interpolated scalp map. Typically, we suggest 64 pixels in each spatial direction. There is also an option to either interpolate or remove bad channels from the images. Interpolation is the preferred option when there is a sufficient number of good channels around each bad channel. If bad channels are removed, there will be “holes” in the resulting images and these holes will be propagated throughout the statistical analysis. A directory is created with the same name as the input dataset. In this directory there will be a subdirectory for each trial type. These directories will contain 3D image files, where the dimensions are space (*x*, *y*) and time (*z*). In the case of averaged data (e.g., an event-related potential-ERP), a single image is placed in each directory. In the case of epoched data, there will be an image for each trial.

#### 3.1.2. Averaging over Time

If the time window of interest is known in advance (e.g., in the case of a well-characterized ERP or event-related field (ERF) peak) one can average over this time window to create a 2D image with just the spatial dimensions.

#### 3.1.3. Time-Frequency Data

**Figure 3:**
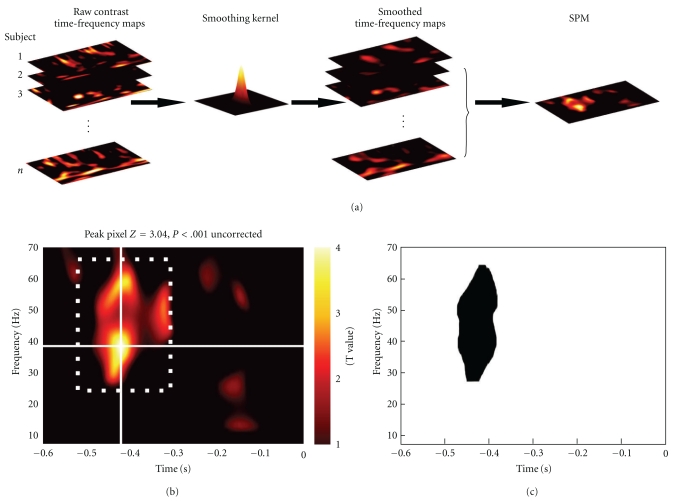
SPM analysis of time × frequency images. (a) Time-frequency images were calculated for each subject and smoothed by convolution with a Gaussian kernel. (b) A *t*-statistic image was calculated from the smoothed time-frequency images and thresholded at *P* < .01 (uncorrected). The location of the peak bin is shown. The white dotted box indicates our illustrative *a priori* window of interest. (c) Statistical test restricted to the window of interest shown in (b) revealed a significant cluster (*P* = .005, cluster-level FWER correction). This figure was adapted with permission from [15].

Although, in principle, topological inference can be done for any number of dimensions, the present implementation in SPM8 is limited to 3 dimensions or less. Thus, when time-frequency features are exported to summary statistic images, it is necessary to reduce the data dimensionality from 4D (space × space × time × frequency) to either a 3D image (space × space × time) or a 2D time-frequency image (time × frequency). This is achieved by averaging either over channels (space × space) or frequencies. Averaging over channels (or as a common special case, selecting one channel) furnishes 2D time-frequency images (Figure 3). 

When averaging over frequencies, one needs to specify the frequency range of interest. The power is then averaged over the specified frequency band to produce channel waveforms. These waveforms are saved in a new time-domain M/EEG dataset. This dataset can be reviewed and further processed in the same way as ordinary time domain datasets (source reconstruction or DCM would not be appropriate because the data features are power or energy [21]). Once this dataset is generated, it is automatically exported to images in the same way as data in the time domain (see above).

#### 3.1.4. Smoothing

The images generated from M/EEG data are generally smoothed prior to second level (i.e., group level) analysis by multidimensional convolution with a Gaussian kernel (standard image smoothing available in SPM). Smoothing is necessary to accommodate spatial/temporal variability over subjects and ensure the images conform to the assumptions of the topological inference approach. The dimensions of the smoothing kernel are specified in the units of the original data: [mm × mm × ms] for space-time, [Hz × ms] for time-frequency images. The guiding principle for deciding how much to smooth is based on the matched filter theorem, which says that the smoothing kernel should match the scale of data features one expects. Therefore, the spatial extent of the smoothing kernel should be more or less similar to the extent of the dipolar patterns expected in the data (probably of the order of a few cm). In practice, one can try smoothing the images with different kernels, according to the principle above; this is a form of scale space search or feature selection. Smoothing in time is not always necessary, as temporal filtering has the same effect. Once the images have been smoothed, one can proceed to the second level analysis.

Figure 2 is a schematic illustrating the construction of (space × space × time) summary-statistic image and the ensuing SPM testing for an effect of faces versus scrambled faces stimuli over subjects. This example highlights the role of topological inference (based on random field theory) to identify significant sensor-time regions that contain a significant condition-specific response. Figure 3 illustrates a typical analysis in time × frequency space using a single channel. These analyses can then be reported directly or used to finesse the subsequent characterisation of the appropriate peristimulus time window and frequency bands in source space.

## 4. Source Analysis

This section focuses on the imaging (or distributed) methods for EEG/MEG source reconstruction in SPM. This approach results in a spatial projection of sensor data into (3D) brain space and considers brain activity as comprising a very large number of dipolar sources spread over the cortical sheet, with fixed locations and orientations. This renders the observation model linear, the unknown variables being the source amplitudes. Given epoched and preprocessed data, the evoked and/or induced activity for each dipolar source can be estimated, for either a short time segment or a wider peristimulus time window. The reconstructed activity is in 3D voxel space and can then be analyzed using mass univariate analysis in SPM, using appropriate summary statistic images over time and/or frequency.

In contrast to PET/fMRI image reconstruction, M/EEG source reconstruction is a nontrivial operation. Often compared to estimating a body shape from its shadow, inferring brain activity from scalp data is mathematically ill-posed and requires prior information such as anatomical, functional, or mathematical constraints to isolate a unique and highly probable solution [27]. Distributed linear models have been around for more than a decade now [28], and the recommended pipeline in SPM for an imaging solution is very similar to common approaches in the field [29, 30]. However, at least three aspects are original and should be emphasized here.

- Based on an empirical Bayesian formalism, the inversion is meant to be generic, in the sense that it can incorporate and estimate the relevance of multiple constraints of a varied nature (i.e., it can reproduce a variety of standard constraints of the sort associated with minimum norm [29], LORETA [30], and other well-known solutions to the inverse problem). The data-driven relevance of different constraints (priors) is established through Bayesian model inversion, and different sets of constraints can be evaluated using Bayesian model comparison [16–18, 31, 32].
- Subject-specific anatomy is incorporated in the generative model of the data, in a fashion that eschews individual cortical surface extraction. The individual cortical mesh is obtained automatically from a canonical mesh in MNI space, providing a simple and efficient way of reporting results in stereotactic coordinates [33].
- SPM uses a Gaussian process model [34, 35] for source reconstruction based on the sample channel × channel covariance of the data over time. Crucially, this means it does not reconstruct one time bin at a time but uses the variance over time to furnish a full spatiotemporal inversion for each time series. This finesses any problems with specifying baselines, because only the variance (change from prestimulus baseline) contributes to the sample covariance and, therefore, the solution. In short, SPM reconstructs changes in source activity (not activity *per* *se*). This becomes important when specifying the time window for inversion (see below).

The M/EEG imaging pipeline is divided into four consecutive steps, which characterize any inverse procedure with an additional step of summarizing the results. In this section, we go through each of the steps that comprise a full inverse analysis.

- Source space modelling.
- Data coregistration.
- Forward computation.
- Inverse reconstruction.
- Summarizing the reconstructed response as an image.

Whereas the first three steps specify the forward or generative model, the inverse reconstruction step is concerned with Bayesian inversion of that model and is the only step that requires the EEG/MEG data.

### 4.1. Getting Started

Everything described below is accessible from the SPM user interface by pressing the “3D Source Reconstruction” button. A new window will appear with a GUI that guides the user through the necessary steps to obtain an imaging reconstruction of their data (see Figure 1(f)). At each step, the buttons not yet relevant for this step will be disabled. At the beginning, only two buttons are enabled: “Load”, which is used to load a preprocessed SPM M/EEG dataset and the “Group inversion” button that will be described below. One can load a dataset that is either epoched with single trials for different conditions, averaged with one ERP/ERF per condition, or grand averaged. An important precondition for loading a dataset is that it should contain sensors and fiducials (see “Section 4.3.”). This will be checked when loading a file and loading will fail if there is a problem. The user should make sure that for each modality in the dataset as indicated by channel types (either EEG or MEG), there is a sensor description. For instance, to load MEG data with some EEG channels that are not actually used for source reconstruction, the type of these channels should be changed to “LFP” (local field potential) or “Other” before trying to load the dataset. Unlike “Other” channels, “LFP” channels are filtered and are available for artefact detection. MEG data converted by SPM from their raw formats will usually contain valid sensor and fiducial descriptions. In the case of EEG, for some supported channel setups (such as extended 10–20 or Biosemi), SPM will provide default channel locations and fiducials that can be used for source reconstruction. Sensor and fiducial descriptions can be modified using the “Prepare” interface (see Appendix B.3).

When a dataset is loaded, the user is asked to give a name to the reconstruction. In SPM, it is possible to perform multiple reconstructions of the same dataset with different parameters. The results of these reconstructions will be stored with the dataset after pressing the “Save” button. They can be loaded and reviewed using the “3D Source Reconstruction” GUI and also with the SPM M/EEG reviewing tool. From the command line, one can access source reconstruction results via the D.inv field of the @meeg object. This field (if present) is a cell array of structures. Each cell contains the results of a different reconstruction. One can navigate among these cells in the GUI, using the buttons in the second row. One can also create, delete, and clear analysis cells. The label provided at the beginning will be attached to the cell for the user to identify it.

### 4.2. Source Space Modelling

**Figure 4:**
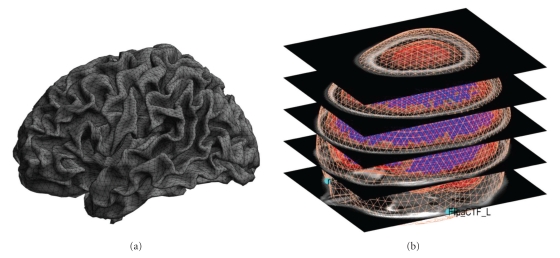
Template meshes used for distributed source imaging. (a) “Normal” cortical template mesh (8196 vertices), left view. The triangular grid shows the representation of the cortical surface used by SPM. (b) All template meshes (cortex, inner skull, outer skull, and scalp) superimposed on the template MRI. Default fiducial locations associated with the template anatomy are displayed in light blue.

After entering the label, the “Template” and “MRI” buttons will be enabled. The “MRI” button creates individual head meshes describing the boundaries of different head compartments based on the subject's structural scan. SPM will ask for the subject's structural image. It might take some time to prepare the model, as the image needs to be segmented as part of computing the nonlinear transformation from individual structural spaces to the template space [5]. The individual meshes are generated by applying the inverse of the spatial deformation field, which maps the individual structural image to the MNI template, to canonical meshes derived from this template [33], Figure 4(b). This method is more robust than deriving the meshes from the structural image directly and can work even when the quality of the individual structural images is low.

In the absence of an individual structural scan, combining the template head model with the individual head shape also results in a fairly precise head model. The “Template” button uses SPM's template head model based on the MNI brain. The corresponding structural image can be found under canonical/single_subj_T1.nii in the SPM directory. When using the template, different things are done depending on whether the data are EEG or MEG. For EEG, the electrode positions will be transformed to match the template head. So even if the subject's head is quite different from the template, one should be able to obtain reasonable results. For MEG, the template head will be transformed to match the fiducials and head shape that come with the MEG data. In this case, having a head shape measurement can be helpful in providing SPM with more data to scale the head correctly. 

Irrespective of whether the “MRI” or “Template” button is used, the cortical mesh describing the locations of possible sources of the EEG and MEG signal is obtained from a template mesh (Figure 4(a)). In the case of EEG, the mesh is used as is, and in the case of MEG it is transformed with the head model. Three cortical mesh sizes are available: “coarse”, “normal”, and “fine” (5124, 8196, and 20484 vertices, resp.). We advise to work with the “normal” mesh. “Coarse” is useful for less powerful computers and “fine” will only work on 64-bit systems with enough main memory. The inner-skull, outer-skull, and scalp canonical surfaces each comprise 2562 vertices, irrespective of the cortical mesh size.

For the purposes of forward computation, the orientations of the sources are assumed to be normal to the cortical mesh. This might seem as a hard constraint at first glance, especially for the “Template” option, where the details of the mesh do not match the individual cortical anatomy. However, in our experience, when a detailed enough mesh is used, the vertices in any local cortical patch vary sufficiently in their orientation to account for any activity that could come from the corresponding brain area; provided the mesh is sufficiently dense. All the (three) mesh resolutions offered by SPM provide sufficient degrees of freedom in this context. When comparing meshes with free and fixed orientation, Henson et al. [36] found the latter to be superior for SPM's default source reconstruction method.

### 4.3. Data Coregistration

For SPM to provide a meaningful interpretation of the results of source reconstruction, it should map the coordinate system in which sensor positions are originally represented to the coordinate system of a structural MRI (MNI coordinates). 

There are two possible ways of coregistering M/EEG data to the structural MRI space.

- A landmark-based coregistration (using fiducials only). The rigid-body transformation matrices (rotation and translation) are computed such that they match each fiducial in the M/EEG space to the corresponding one in MRI space. The same transformation is then applied to the sensor positions.
- Surface matching (between some head shape in M/EEG space and some MRI-derived scalp tessellation).

For EEG, the sensor locations can be used instead of the head shape. For MEG, the head shape is first coregistered with MRI space; the inverse transformation is then applied to the head model and the mesh. Surface matching is performed using an iterative closest point algorithm (ICP). The ICP algorithm [37] is an iterative alignment algorithm that works in three phases.

- Establish correspondence between pairs of features in the two structures that are to be aligned, based on proximity.
- Estimate the rigid transformation that best maps the first member of the pair onto the second.
- Apply that transformation to all features in the first structure. These three steps are then reapplied until convergence. Although simple, the algorithm works quite effectively when given a good initial estimate.

In practice, after pressing the “Coregister” button one needs to specify the points in the MRI that correspond to the M/EEG fiducials. If more than three fiducials are available (which may happen for EEG as, in principle, any electrode can be used as a fiducial), the user is asked at the first step to select the fiducials to use. It is possible to select more than three, but not less. Then for each M/EEG fiducial selected, the user is asked to specify the corresponding position in the MRI in one of three ways.

- “Select”—locations of some points such as the commonly used nasion and preauricular points and also CTF-recommended fiducials for MEG are hard-coded in SPM. If an M/EEG fiducial corresponds to one of these points, the user can select this option and then select the correct point from a list.
- “Type”—here it is possible to enter the MNI coordinates for the fiducial (1 × 3 vector). If the fiducial is not in the SPM hard-coded list, it is advised to carefully find the correct point on either the template image or on the subject's own image registered to the template. This can be done by opening the image using SPM's image display functionality. One can then record the MNI coordinates and use them in subsequent coregistration, using the “type” option.
- “Click”—the user is presented with a structural image and can click on the correct point. This option is good for “quick and dirty” coregistration or to try out different options.

After specifying the fiducials, the user is asked whether to use the head shape points if they are available. For EEG this is advised. For MEG, the head model is based on the subject's MRI, and precise information about the fiducials is available (e.g., from a MRI with fiducials marked by vitamin E capsules); using the head shape might actually do more harm than good.

**Figure 5:**
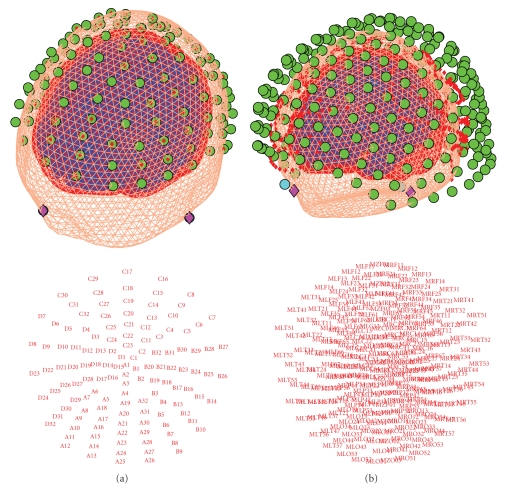
Examples of coregistration display (appears after the co-registration step has been completed). Top row shows the 3D outcome of the co-registration, while the bottom row shows the sensor arrangement in 2D with corresponding labels. (a) EEG data (128 Biosemi system) from the multimodal face perception experiment available from the SPM website. EEG sensor locations have been adjusted to fit the scalp surface. (b) MEG data (275-channel CTF system) from the same experiment.

The results of the coregistration are presented in SPM's graphics window (see Figure 5). It is important to examine the results carefully before proceeding. The top panel shows the scalp, the inner skull, and the cortical mesh, with the sensors and the fiducials. For EEG one should make sure that the sensors are on the scalp surface. For MEG one should check that the head position, in relation to the sensors, makes sense and the head does not, for instance, protrude outside the sensor array. In the bottom panel, the sensor labels are shown in topographical array. One should check that the top labels correspond to anterior sensors, bottom to posterior, left to left, and right to right and also that the labels are where expected topographically (e.g., that there is no shift when matching positions to channels).

### 4.4. Forward Computation

This refers to computing, for each dipole on the cortical mesh, the effect it would have on the sensors. The result is an *N* × *M* matrix where *N* is the number of sensors and *M* is the number of mesh vertices (chosen from several options at a previous step). This matrix can be quite large and is therefore stored in a separate MAT-file (whose name starts with “SPMgainmatrix”). This file is written to the same directory as the dataset. Each column in this matrix is a so-called “lead field”, corresponding to one mesh vertex. The lead fields are computed using the “forward” toolbox, which SPM shares with FieldTrip (see Oostenveld et al., this issue). This computation is based on Maxwell's equations and makes assumptions about the physical properties of the head. There are different ways to specify these assumptions which are known as “forward models”.

**Figure 6:**
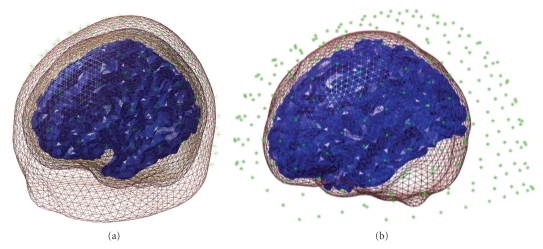
Examples of forward model display (appears after the forward modelling step has been completed). The figure includes the cortical mesh, the sensor locations and the other layers used to compute the lead-field matrix. (a) EEG data (128 Biosemi system) from the multimodal face perception experiment available from the SPM website. This figure shows the head model that was used to compute the BEM forward solution for these data. (b) MEG data (275-channel CTF system) from the same experiment. This figure shows the head model that was used to compute the realistic single shell solution for these data.

The “forward” toolbox supports different forward models. After pressing the “Forward Model” button (which should be enabled after successful coregistration), the user has a choice of several head models, depending on the modality of the data. In SPM8, we recommend using a “single shell” model [38] for MEG and “EEG BEM” (Boundary Elements Model [39–43]) for EEG. One can also try other options and compare them using their model evidence ([36], see below). The first time the EEG BEM option is used with a new structural image (and also the first time the “Template” option is used) a lengthy computation will take place that prepares the BEM model based on the head meshes. The BEM will then be saved in a large MAT-file with ending “_EEG_BEM.mat” in the same directory as the structural image (this is the “canonical” subdirectory of SPM for the template). When the head model is ready, it will be displayed in the graphics window, with the cortical mesh and sensor locations, for verification (Figure 6). The actual lead field matrix is computed at the beginning of the next step and saved. This is a time-consuming step, particularly for high-resolution meshes. The lead field file will be used for all subsequent inversions, if the coregistration and the forward model are not changed.

### 4.5. Inverse Reconstruction

The inverse reconstruction is invoked by pressing the “Invert” button. The first choice one gets is between “Imaging”, “VB-ECD”, and “DCM”. For reconstruction based on an empirical Bayesian approach (to localize evoked responses, evoked power, or induced power) one should press the “Imaging” button. The other options are explained in greater detail below. When there are several conditions (trial types) in the dataset, then the next choice is whether to invert all the conditions together or to choose a subset. If one is planning a statistical comparison between a set of conditions, one should invert all of them together. After selecting the conditions one gets a choice between “Standard” and “Custom” inversion. For “Standard” inversion, SPM will start the computation with default settings. These correspond to the multiple sparse priors (MSP) algorithm [17], which is then applied to the whole time series.

To fine tune the parameters of the inversion, the “Custom” option can be chosen. There will then be a possibility to choose among several types of inversion, differing in terms of their hyperpriors (priors on priors or constraints): IID—equivalent to classical minimum norm [29], COH—smoothness prior similar to methods such as LORETA [30], or multiple sparse priors (MSP) [17]. The latter gives the most plausible results and has been shown to have greater model evidence in relation to other priors [36].

One can then choose the time window that will be used for inversion. Based on our experience, we recommend the time window be limited to periods in which the activity of interest is expressed. The reason is that if irrelevant high-amplitude activity is included, source reconstruction will focus on reducing the error for reconstructing this activity and might suppress the responses of interest. There is also an option to apply a Hanning taper to the time series to down-weight possible baseline noise at the beginning and end of the trial. The next option is to prefilter the data. This is mainly for focusing on certain temporal scales during reconstruction (e.g., alpha band for ERPs or gamma for faster induced responses or sensory-evoked responses). The next option allows for extra source priors. This makes it possible to integrate prior knowledge from the literature or from fMRI/PET/DTI into the inversion [44]. Here, one can just provide a thresholded statistical image and SPM will generate the priors based on its suprathreshold clusters. Custom priors are not a “hard” way to restrict the solution. They will only be used when leading to a solution with higher model evidence. A “hard” restriction of the inverse solution is provided by the next option.

Here, one can restrict solutions to particular brain areas by loading (or specifying) a MAT-file with a *K* × 3 matrix, containing MNI coordinates of the areas of interest. This option may seem strange initially; as it may seem to overly bias the source reconstruction. However, in the Bayesian inversion framework, it is possible to compare different inversions of the same data using Bayesian model comparison. By limiting the solutions to particular brain areas, one can greatly simplify the model, and if this simplification appropriately captures the sources generating the response, then the restricted model will have higher model evidence than the unrestricted one. If, however, the restricted sources cannot account for the data, the restriction will result in a worse model fit and the unrestricted model might be better (note that for model comparison to be valid, all settings that affect the data, like the time window and filtering, should be identical).

SPM8 imaging source reconstruction also supports multimodal datasets. These are datasets that have both EEG and MEG data from a simultaneous recording. Datasets from the Elekta/Neuromag Vectorview MEG system, which has two kinds of MEG sensors, are also treated as multimodal. If the dataset is multimodal, a dialogue box will appear asking one to select the modalities for source reconstruction from a list. When selecting more than one modality, multimodal fusion will be performed. This option uses a heuristic to rescale the data from different modalities so that they can be fused [45]. 

**Figure 7:**
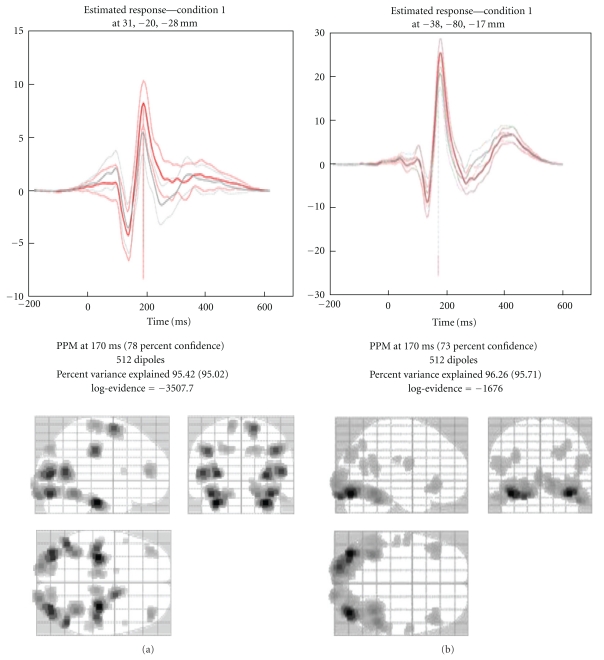
Display of the estimated distributed solution for evoked responses. The top panel shows the time course of the source having maximal activity while the bottom panel shows the maximum intensity projections (MIP) at the time of maximum activation. (a) EEG data from the multimodal face perception experiment available from the SPM website. (b) MEG data from the same experiment. Time course in red is for the face stimuli while the light grey is for scrambled faces. The lighter red and gray lines indicate 90% confidence intervals.

Once the inversion is complete, the time course of the source with maximal activity is presented in the top panel of the graphics window (see Figure 7). The bottom panel shows the maximum intensity projection (MIP) at the time of the maximum activation. The log-evidence, which can be used for model comparison as explained above, is also shown. Note that not all of the output of the inversion is displayed. The full output consists of time courses for all the sources and conditions for the entire time window. It is possible to view more of these results using the controls in the bottom right corner of the 3D GUI. These allow one to focus on a particular time, brain area, and condition. One can also display a movie of the evolution of source activity.

### 4.6. Summarizing the Reconstructed Response as an Image

SPM allows one to create summary statistic images in terms of contrasts (mixtures of parameter or activity estimates) over time and frequency. These are in the form of 3D NIfTI images, so that one can proceed to GLM-based statistical analysis in the usual way (at the between-subject level). This entails summarizing the trial- and subject-specific responses with a single 3D image in source space and involves specifying a time-frequency window for each contrast image. This is a flexible and generic way of specifying the data features one wants to make an inference about (e.g., gamma activity around 300 ms or average response between 80 and 120 ms). The contrast is specified by pressing the “Window” button. The user will then be asked about the time window of interest (in ms, peristimulus time). It is possible to specify one or more time segments (separated by a semicolon). To specify a single time point the same value can be repeated twice. The next prompt pertains to the frequency band. To average the source time course one can simply leave this at the default of zero. In this case, the window will be weighted by a Gaussian function. In the case of a single time point, this will be a Gaussian with 8 ms full width half maximum (FWHM). If one specifies a particular frequency or a frequency band, then a series of Morlet wavelet projectors will be generated, summarizing the energy in the time window and frequency band of interest.

There is a difference between specifying a frequency band of interest as zero, as opposed to specifying a wide band that covers the whole frequency range of the data. In the former case, the time course of each dipole is averaged over time, weighted by a Gaussian. Therefore, if within the selected time window this time course changes polarity, the activity can average out and even a strong response can produce a value of zero. In the latter case, the power is integrated over the whole spectrum ignoring phase, and this would be equivalent to computing the sum of squared amplitudes in the time domain.

**Figure 8:**
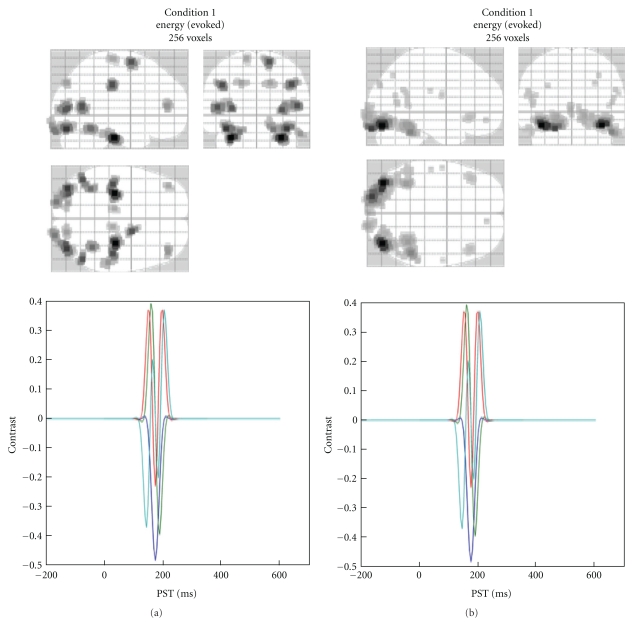
Display of the estimated distributed solution for evoked power in a specific frequency band. The same can be obtained for induced power and for each trial. The top panel shows the maximum intensity projections (MIP) while the bottom panel shows the applied time-frequency contrast. (a) EEG data from the multimodal face perception experiment available from the SPM website. (b) MEG data from the same experiment. Evoked power was computed between 150 and 200 ms, for frequencies between 1 and 20 Hz.

Finally, if the data file is epoched rather than averaged, there is a choice between “evoked”, “induced”, and “trials”. The projectors generated at the previous step can either be applied to each trial and the results averaged (induced) or applied to the averaged trials (evoked). Thus, it is possible to localize induced activity that has no phase locking to the stimulus. It is also possible to focus on frequency content of the ERP using the “evoked” option. Clearly the results will not be the same. The projectors specified (bottom panel of Figure 8) and the resulting MIP (top panel) will be displayed when the operation is completed. The “trials” option makes it possible to export an image per trial, which is useful for performing parametric within-subject analyses (e.g., looking for the correlates of reaction times).

**Figure 9:**
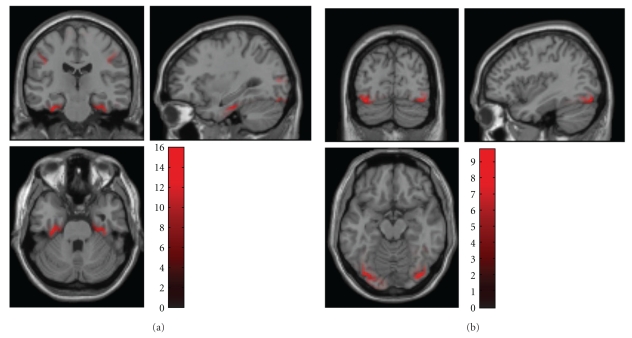
Axial, sagittal, and coronal views of the contrast image shown in Figure 8, projected into MNI voxel space and superimposed on the template structural MRI image. (a) EEG data from the multimodal face perception experiment available from the SPM website. (b) MEG data from the same experiment. The intensity was normalised to the mean over voxels to reduce intersubject variance.

The values of the exported images are normalized to reduce between-subject variance. Therefore, for best results one should export images for all the time windows and conditions that will be included in the same statistical analysis together. Note that the images exported from the source reconstruction are a little peculiar because of smoothing from a 2D cortical sheet into 3D volume (Figure 9). SPM's statistical machinery has been optimized to deal with these peculiarities and ensure sensible results.

In what follows, we consider some auxiliary functions associated with source reconstruction.

#### 4.6.1. Rendering Interface

By pressing the “Render” button one can open a new GUI window, which displays a rendering of the inversion results on the brain surface. One can rotate the brain, focus on different time points, run a movie, and compare the predicted and observed scalp topographies and time series. A useful option is the “virtual electrode”, which allows one to extract the time course from any point on the mesh and form the MIP at the time of maximum activation at this point. An additional tool for reviewing the results is available in the SPM M/EEG reviewing tool.

#### 4.6.2. Group Inversion

A problem encountered with MSP inversion is that it sometimes produces solutions that are so focal in each subject that the spatial overlap between the activated areas across subjects is not sufficient to yield a significant result at the between-subject level. This could be finessed by smoothing, but smoothing compromises the spatial resolution and thus subverts the main advantage of using an inversion method that can produce focal solutions. The more principled solution is to tell the model that the same distributed brain system has been engaged in all subjects or sessions (by design). This is simple to do using a hierarchical extension of the MSP method [46] that effectively ensures the activated sources are the same in all subjects (only the time course of activation is allowed to vary over subjects). We showed that this modification makes it possible to obtain significance levels close to those of nonfocal methods such as minimum norm, while preserving accurate spatial localization. Group inversion can yield much better results than individual inversions because it introduces an additional constraint for the ill-posed inverse problem, namely, that the responses in all subjects should be explained by the same set of sources. It is the inversion method of choice, when analyzing an entire study with subsequent topological inference on the contrast images.

Operationally, group inversion involves computing prior spatial covariances in source space that are common to all subjects. This rests on realigning the sensor-level data to pool sample covariances over subjects. In principle, this is straightforward because the linear mappings from each subject's sensors to a canonical set of cortical sources imply there is a unique linear mapping from one subject's montage to another; in other words, we can compute what we would have seen if one subject had been studied with the montage of another subject. However, in practice, the requisite realignment is a little more difficult because the “average” montage must converse information from all subjects. Imagine that two subjects have been studied with a single electrode and that the lead fields of these two electrodes are orthogonal. This means that realigning the sensor from one subject with the other would lose all the information from the subject being realigned. What we seek is an average sensor that captures the signals from both subjects in a balanced way. This can be achieved by iteratively solving a set of linear equations under the constraint that the mutual information between the average (realigned) sensor data and each subject's data is maximized. SPM8 uses a recursive (generalised) least squares scheme to do this.

Group inversion can be started by pressing the “Group inversion” button right after opening the 3D source reconstruction GUI. The user is asked to specify a list of M/EEG datasets to invert together. Then one is asked to coregister each of the files and specify all the inversion parameters in advance. It is also possible to specify contrasts in advance. Then the inversion will proceed by computing the inverse solution for all the files and will write out the output images. The results for each subject are saved in the header of the corresponding input file. It is possible to load this file into the 3D GUI, after inversion, and explore the results as described above.

#### 4.6.3. Batching Source Reconstruction

One can also run imaging source reconstructions using the matlabbatch tool. It can be accessed by pressing the “Batch” button in the main SPM window and then going to “M/EEG source reconstruction” under “SPM” and “M/EEG”. There are three separate tools here for building head models, computing the inverse solution and creating contrast images. This makes it possible to generate images for several different contrasts from the same inversion. All the three tools support multiple datasets as inputs. Group inversion is used automatically for multiple datasets. 

This completes our review of distributed source reconstruction. Before turning to the final section, we consider briefly the alternative sort of source space model, which is much simpler but, unlike the cortical mesh described in this section, leads to a nonlinear forward model parametrisation.

## 5. Localization of Equivalent Current Dipoles

This section describes source reconstruction based on Variational bayesian equivalent current dipoles (VB-ECDs) [19]. 3D imaging (or distributed) reconstruction methods consider all possible source locations simultaneously, allowing for large and distributed clusters of activity. This is to be contrasted with “equivalent current dipole” (ECD) approaches, which rely on two hypotheses.

- Only a few (say less than ~5) sources are active simultaneously, and that
- those sources are focal.

This leads to the ECD forward model, where the observed scalp potential is explained by a handful of discrete current sources; that is, dipoles, located inside the brain volume. In contrast to imaging reconstruction, the number of ECDs considered in the model, that is, the number of active locations, has to be defined *a priori*. This is a crucial step, as the number of sources considered defines the ECD model. This choice should be based on empirical knowledge about the brain activity observed or any other source of information (e.g., by looking at the scalp potential distribution). Note that the number of ECDs can be optimised *post hoc* using model comparison (see below). In general, each dipole is described by six parameters: three for its location, two for its orientation, and one for its amplitude. To keep the inverse problem overdetermined, the number of ECDs therefore must not exceed the number of channels divided by 6, and preferably should be well below this threshold. Once the number of ECDs is fixed, a nonlinear Variational Bayesian scheme is used to optimise the dipole parameters (six times the number of dipoles) given the observed potentials.

Classical ECD approaches use a simple best fitting optimisation using “least square error” criteria. This leads to relatively simple algorithms but presents a few drawbacks.

- Constraints on the dipoles are difficult to include in the framework.
- Noise cannot be properly taken into account, as its variance should be estimated alongside the dipole parameters.
- It is difficult to define confidence intervals on the estimated parameters, which could lead to overconfidence in the results.
- Models with different numbers of ECDs cannot be compared, except through their goodness-of-fit, which can be misleading. As adding dipoles to a model will necessarily improve the overall goodness of fit, one could erroneously be tempted to use as many ECDs as possible and to perfectly fit the observed signal.

However, using Bayesian techniques, it is possible to circumvent all of the above limitations of classical approaches. Briefly, a probabilistic generative model is built, providing a likelihood model for the data. This assumes an independent and identically distributed normal distribution for the errors, but other distributions could be specified. The model is completed by priors on the various parameters, leading to a Bayesian forward model, which allows the inclusion of user-specified prior constraints. 

An iterative Variational Bayesian scheme is then employed to estimate the posterior distribution of the parameters (in fact the same scheme used for distributed solutions). The confidence interval on the estimated parameters is therefore directly available through the posterior variance of the parameters. Crucially, in a Bayesian context, different models can be compared using their evidence. This model comparison is superior to classical goodness-of-fit measures, because it takes into account the complexity of the models (e.g., the number of dipoles) and, implicitly, uncertainty about the model parameters. VB-ECD can therefore provide an objective and accurate answer to the question: would this dataset be better modelled by two or three ECDs? We now describe the procedure for using the VB-ECD approach in SPM8.

The engine calculating the projection (lead field) of the dipolar sources to the scalp electrodes comes from FieldTrip and is the same for the 3D imaging or DCM. The head model should thus be prepared the same way, as described in the previous section. For the same data set, differences between the VB-ECD and imaging reconstructions are, therefore, only due to the reconstruction chosen.

### 5.1. VB-ECD Reconstruction

After loading and preparing the head model, one should select the VB-ECD option after pressing the “Invert” button in the “3D source reconstruction” window. The user is then invited to fill in information about the ECD model and click on buttons in the following order.

- Indicate the time bin or window for the reconstruction. Note that the data will be averaged over the selected time window. VB-ECD will thus always be calculated for a single scalp topography.
- Enter the trial type(s) to be reconstructed. Each trial type will be reconstructed separately.
- Add a single (i.e., individual) dipole or a pair of symmetric dipoles to the model.
- Select “Informative” or “Noninformative” location priors. “Non-informative” invokes flat priors over the brain volume. With “Informative”, one can enter the *a priori* location of the source (for a symmetric pair of dipoles, only one set of dipole coordinates is required).
- At this point, it is possible to go back and add more dipole(s) to the model, or stop adding dipoles.
- Specify the number of iterations. These are repetitions of the fitting procedure with different initial conditions. Since there are multiple local maxima in the objective function, multiple iterations are necessary to ensure good results, especially when non-informative location priors are chosen.

**Figure 10:**
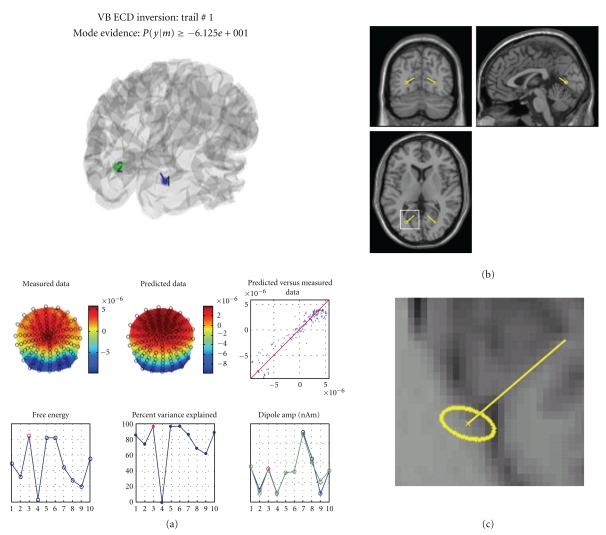
VB-ECD solution illustrated here on EEG data from the multimodal face perception experiment available from the SPM website. A symmetric dipole pair was fitted to the topography of the difference between faces and scrambled faces averaged between 170 and 180 ms. (a) The upper part shows the dipole location through the transparent cortical mesh; the middle part shows the correspondence between observed and predicted scalp data in two ways (topographies and dot plot); the bottom part shows the free energy, the explained variance, and the estimated dipole amplitude (from left to right) obtained from each of the ten repetitions of the procedure with different initial locations. Results correspond to the one with highest free energy (red point). (b) Orthogonal views of the brain with dipole locations obtained from the solution with highest free energy. (c) Enlarged fragment of the axial image shown by white square in (b). The ellipse shows the 95% confidence volume for dipole location.

The routine then proceeds with the VB optimization scheme to estimate the model parameters. There is a graphical display of intermediate results. When the best solution is selected, the model evidence will be shown at the top of the SPM graphics window (see Figure 10(a)). This can be used to compare solutions with different priors or number of ECDs. Results of the inversion are saved to the data structure and displayed in the graphics window.

#### 5.1.1. Result Display

The VB-ECD results can be displayed again by pressing the “dip” button, located under the “Invert” button that will be enabled after computing VB-ECD solution. In the upper part, the three main figures display orthogonal views of the brain with the dipole location and orientation superimposed (see Figure 10(b)). The location confidence interval is reported by the dotted ellipse around the dipole location (Figure 10(c)). The lower left table displays the current dipole location, orientation (Cartesian or polar coordinates), and amplitude in various formats. The lower right table allows for the selection of trial types and dipoles. Display of multiple trial types and multiple dipoles is also possible. The display will centre itself on the average location of the dipoles. 

This completes our discussion of source reconstruction. The previous sections introduced distributed and ECD solutions based on forward models mapping from sources to sensors. These models are not constrained to produce physiologically plausible neuronal activity estimates and ignore the neuronal coupling among different dipolar sources in generating observed sensor signals. In the final section, we turn to dynamic causal modelling (DCM), which effectively puts a neuronal model underneath the electromagnetic forward models considered above. Usually, source reconstruction (imaging or ECD) is used to answer questions about the functional anatomy of evoked or induced responses, in terms of where sources have been engaged. This information is generally used to specify the location priors of sources in DCM.

## 6. Dynamic Causal Modelling for M/EEG

Dynamic causal modelling (DCM) is based on an idea initially developed for fMRI data [8]. Briefly, measured data are explained by a network model consisting of a few sources, which are dynamically coupled (cf. spatiotemporal dipole modelling introduced by Scherg and colleagues [47, 48]). This network model is inverted using the same Variational Bayesian scheme used for source reconstruction. Model inversion furnishes the model evidence (used to search model spaces or hypotheses) and the posterior density on model parameters (used to make inferences about connections between sources or their condition-specific modulation), under the model selected. David et al. [20] extended the DCM idea to modelling ERPs. At its heart DCM for ERP (DCM-ERP) is a source reconstruction technique, and for the spatial domain we use exactly the same forward model as the approaches in previous sections. However, what makes DCM unique is that it combines the spatial forward model with a neurobiologically informed temporal forward model, describing the connectivity among sources. This crucial ingredient not only makes the source reconstruction more robust, by implicitly constraining the spatial parameters, but also allows inference about connectivity architectures.

For M/EEG data, DCM can be a powerful technique for inferring (neuronal) parameters not observable with M/EEG directly. Specifically, one is not limited to questions about source strength, as estimated using a source reconstruction approach, but can test hypotheses about connections between sources in a network. As M/EEG data are highly resolved in time, as compared to fMRI, precise inferences about neurobiologically meaningful parameters (e.g., synaptic time constants) are possible. These relate more directly to the causes of the underlying neuronal dynamics. In the recent years, several variants of DCM for M/EEG have been developed. DCM for steady state responses (DCM-SSR) [22, 49, 50] uses the same neural models as DCM-ERP to generate predictions for power spectra and cross-spectra measured under steady state assumptions. There are also (phenomenological) DCMs that model specific data features, without an explicit neural model. DCM for induced responses (DCM-IR) [21] models event-related power dynamics (time-frequency features). DCM for phase coupling (DCM-PHA) [23] models event-related changes in phase relations between brain sources: DCM-PHA can be applied to one frequency band at a time. Presently, all M/EEG DCMs share the same interface, as many of the variables that need to be specified are the same for all four approaches. Therefore, we will focus on DCM for evoked responses and then point out where the differences to the other DCMs lie. 

In this section, we only provide a procedural guide for the practical use of DCM for M/EEG. For the scientific background, the algorithms used or how one would typically use DCM in applications, we recommend the following. A general overview of M/EEG DCMs can be found in [51]. The two key technical contributions for DCM-ERP can be found in [20, 52]. Tests of interesting hypotheses about neuronal dynamics are described in [53, 54]. Other examples of applications demonstrating the kind of hypotheses testable with DCM can be found in [55, 56]. Another good source of background information is the recent SPM book [57] where parts 6 and 7 cover not only DCM for M/EEG but contextualise DCM with related research from our group. DCM-IR is covered in [21, 58], DCM-SSR in [22, 49, 50], and DCM-PHA in [23]. 

### 6.1. Overview

The goal of DCM is to explain measured data (such as evoked responses) as the output of an interacting network consisting of several areas, some of which receive input (i.e., the stimulus). The differences between evoked responses, measured under different conditions, are modelled as a modulation of specified DCM parameters; for example, cortico-cortical connections [20]. The implicit model of evoked responses makes hypotheses about connectivity directly testable. For example, one can ask whether the difference between two evoked responses can be explained by top-down modulation of early areas [55]. Importantly, because model inversion is implemented using a Bayesian approach, one can compare Bayesian model evidences. These can be used to compare alternative, equally plausible, models and select the best [9–11].

DCM for evoked responses takes the spatial forward model into account. This makes DCM-ERP a spatiotemporal model of the full data set (over channels and peristimulus time). Alternatively, one can describe DCM as a spatiotemporal source reconstruction algorithm, which uses additional temporal constraints given by neural mass dynamics and long-range effective connectivity. This is achieved by parameterising the lead field, that is, the spatial projection of source activity to the sensors. In the current version, this can be done using two different approaches. The first assumes that the lead field of each source is modelled by a single equivalent current dipole (ECD) [52]. The second models each source as a “patch” of dipoles on the grey matter sheet [59]. This spatial model is complemented by a model of the temporal dynamics of each source. Importantly, these dynamics not only describe how the intrinsic source dynamics evolve over time, but also how a source reacts to external input, from subcortical areas (stimulus) or from other cortical sources.

The GUI allows one to enter all the information necessary for specifying a spatiotemporal DCM for a given data set. To fit multiple models, we recommend using a batch script. An example of such a script can be found in the man/example_scripts folder of the distribution.

### 6.2. Getting Started

**Figure 11:**
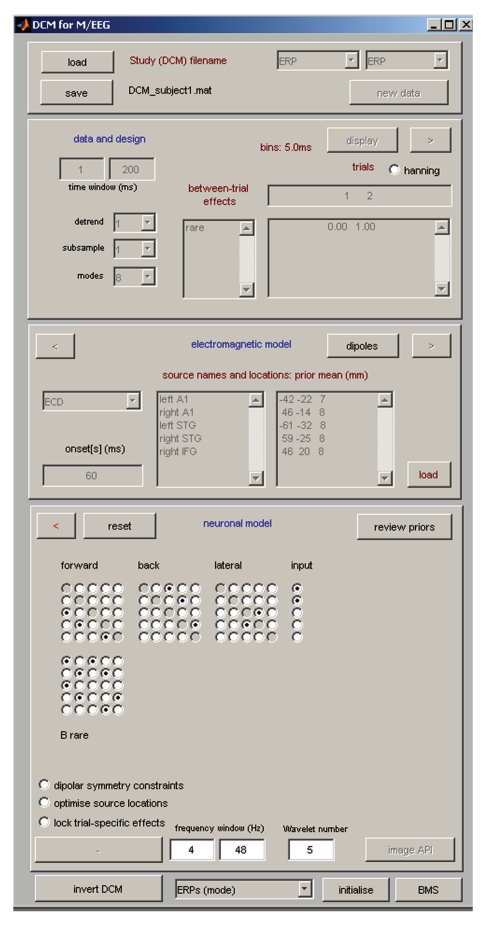
DCM for M/EEG graphical user interface. The configuration shown corresponds to the example DCM for mismatch negativity experiment available from the SPM website; see text for additional details.

The button for calling the DCM GUI is found in the menu window of SPM. When pressing the button, the GUI pops up (Figure 11). The GUI is partitioned into five parts, going from the top to the bottom. The first part deals with loading and saving existing DCMs, and selecting the type of model. The second part is about selecting data, the third is for specification of the spatial forward model, and the fourth is for specifying neuronal connections. The last row of buttons calls the DCM inversion and results display.

Data selection and model specification must be performed in a fixed order (data selection > spatial model > connectivity model). This order is necessary because there are dependencies among the three parts that would otherwise be hard to resolve. At any time, it is possible to switch back and forth from one part to the next. Also, within each part, information can be specified in any order.

### 6.3. Load, Save, and Select Model Type

The buttons at the top part of the GUI allow one to load an existing DCM or save the current one. In general, saving is possible during model specification at any time. There are two drop-down boxes in this part of the DCM-GUI. The one on the left is for switching between different DCM variants. The default is ERP which is the DCM for evoked responses described here. Currently, there are three additional options: IND, SSR, and PHA as mentioned above. The menu on the right-hand side is for choosing the neuronal model. Currently, there are four model types. The first is ERP which is the standard model described in most of the application papers; for example [55]. The second is SEP which uses a variant of this model; however, priors on the neuronal dynamics make them faster to model early evoked responses [60]. The third is NMM which is a nonlinear (conductance-based) neural mass model [61]. The fourth is a mean field model MFM which is also nonlinear and is based on a second-order approximation to population dynamics [62]. Finally, data can be loaded using the “new data” button. The data can be either averaged or epoched EEG or MEG. For DCM-ERP, epoched data will be averaged to produce evoked potentials or fields.

#### 6.3.1. Data and Design

This part deals with selecting and refining the data and modelling between trial effects. On the right-hand side of the DCM GUI, there are three text boxes that specify between-trial effects. These are the effects that are mediated by changing connection strengths. The top box should contain the indices of conditions to include in the model. For example, to model the second and third evoked response contained within a dataset, 2 and 3 should be specified. The indices correspond to the order, which can be specified by the user (see Appendix D.7). If the two evoked responses, for some reason, are in different files, these files need to be merged prior to DCM. Below the condition selection box, there is a box for specification of effects. This is used to define different options for modelling the experimental effects (i.e., the differences between conditions). For example, if trial 1 is the standard and trial 2 is the deviant response in an oddball paradigm, one can use the standard as the baseline and model the differences by modulations of the connections that are necessary to fit the deviant. To do this the effect should be specified as [0 1]. Alternatively, if the effect is specified as [−1 1], then the baseline will be the average of the two conditions and the same factor will be subtracted from the baseline connections to model the standard and added to the connections to model the deviant. The latter option is perhaps not optimal for an oddball paradigm but might be suitable for other paradigms where there is no clear “baseline condition”. When modelling three or more evoked responses, one can model modulations of connection strength over multiple conditions as two effects relative to the first evoked response. However, one can also choose to couple the connection strength over conditions by imposing a relationship on how this connection changes. For example a single linear effect, over three trials or conditions, can be specified as [−1 0 1]. This can be useful when one wants to add constraints on how connections (or other DCM parameters) change. A compelling example of this can be found in [63]. For each experimental effect specified, one later selects the connections in the model that it affects (see below).

The leftmost textbox can be used to define names for the experimental effects (e.g., “oddball”). Further to the left there are several more controls whose purpose is refining the data before modelling. Under “time window (ms)” one has to enter the peristimulus times to model, for example, 1 to 200 ms. One can also choose whether to model the mean or drifts of the data at the sensor level. Under “detrend” one can select the number of discrete cosine transform terms to use to model low frequency drifts (selecting 1 means that just the mean will be removed). In the “subsample” option, one may choose to downsample the data before computing the ERP. This subsampling is not proper down sampling but decimation, so it is not advised to use it routinely. If necessary, it is preferable to down-sample the data during pre-processing. In DCM, we use a projection of the data to a subspace (mixtures of channels) to reduce the amount of data and suppress noise. This spatial projection is described in [54]. One can select the number of modes: the default is 8. One can also choose to window the data, in peristimulus time, with a Hanning window (radio button). This will reduce the influence of the beginning and end of the time series, which might be noisy or not captured by the idealised responses used to predict observed data.

Once satisfied with data selection, the projection and the detrending terms, the user can click on the “>” (forward) button to go to the next stage, electromagnetic model. From this, the red “<” button can be used, if necessary, to get back to the data and design specification.

#### 6.3.2. Electromagnetic Model

Presently, there are three options for how to model evoked responses spatially. The first is to use a single equivalent current dipole (ECD) for each source, the second is to use a patch on the cortical surface (IMG), and the third (LFP) is to not use a spatial model at all (and assume that each channel samples a source with unknown gain). In all three cases, it is necessary to enter the source names (one name in one row). For ECD and IMG, the prior source locations (in mm in MNI coordinates) must be specified. Note that by default DCM uses uninformative priors on dipole orientations, but tight priors on locations. This is because M/EEG data contains limited information about location but supports precise estimates of orientation [64, 65]. This means each dipole stays in its designated area and retains its meaning in terms of anatomical designation. The prior location for each dipole can be found either by using available anatomical knowledge or by relying on source reconstructions (see the previous sections). Also note that the prior location does not need to be overly exact, because the spatial resolution of M/EEG, depending on location, can be on a scale of several centimetres [65, 66]. It is also possible to load the prior locations from a file (“load”). The locations can be visualized by pressing “dipoles”. 

An “onset” parameter determines when the stimulus, presented at 0 ms peristimulus time, is assumed to activate the cortical area to which it is connected. In DCM, we usually do not model small early responses, but start modelling at the first large deflection. Because the propagation of the stimulus impulse through the input nodes causes a delay, we find that the default value of 60 ms onset time is a good value for many evoked responses where the first large deflection (population response) is seen around 100 ms. However, this value is a prior; that is, the inversion routine can optimise it. The prior mean should be chosen according to the specific responses of interest. This is because the time until the first large deflection is dependent on the paradigm or the modality; for example, audition or vision, cortical or subcortical, and so forth. Changing the onset prior might have an effect on how the data are fitted. This is because the onset time has strongly nonlinear effects (a delay) on the predicted responses, which might induce local minima in the solution space, for different prior values. It is also possible to type several numbers in this box (identical or not). Each value invokes its own separate input, whose timing will be optimised separately. These inputs can be connected to the same or different sources of the model. This can be useful, for instance, for modelling a paradigm with combined auditory and visual stimulation.

To proceed to the next model specification stage, hit the “>” (forward) button and proceed to the “Neuronal model”. If this is the first estimation and source reconstruction has not been previously done with the same dataset, DCM will build a spatial forward model. The steps here are the same as described in the “3D source reconstruction” section above.

#### 6.3.3. Neuronal Model

This section is the most critical for model specification. In DCM, one usually specifies a series of models (model space) for model comparison. For the model comparison to be valid, the models should only differ in their connectivity; that is, in the parameters specified in this part. DCM-ERP (as well as DCM-SSR) makes it possible to compare models with different sources. However, because of the data projection used (see above and [54]), this should be done by specifying all the sources in “Electromagnetic model” and then leaving the sources not participating in a particular neuronal model unconnected. 

In this part of the GUI, there are five (or more) matrices, which are specified by radio button switches. The first three are the connection strength (*A*) matrices. For ERP and SEP models, there are three types of connections: forward, backward, and lateral. For NMM and MFM models the corresponding types are excitatory, inhibitory, and mixed (excitatory and inhibitory) connections, respectively. These matrices encode connections from source regions to target regions. For example, switching on the element **(2, 1)** (i.e., second row, first column) in the intrinsic forward connectivity matrix means that a forward connection from area 1 to 2 is enabled (can take nonzero values). This is basically an adjacency matrix for those familiar with graph theory. Some people find the meaning of each element slightly counterintuitive, because the column index corresponds to the source region, and the row index to the target region. This convention is motivated by the direct correspondence between the matrices in the GUI and connectivity matrices in DCM equations, and is probably intuitive to anyone familiar with matrix algebra.

The one or more inputs (onsets) specified previously can go to any source or to multiple sources. Receiving sources can be specified by selecting indices in the input (*C*) matrix. The number of columns in this matrix corresponds to the number of inputs specified previously. For a single input, *C* is a column vector. The bottom set of matrices (*B*-matrices) specify gain modulations of connection strengths as set in the *A*-matrices. These modulations are specified by the experimental effects described above. For example, for two evoked responses and experimental effect specified as [0 1], DCM explains the first response by using the *A*-matrix only. The 2nd response is modelled by modulating the connections specified by the *B*-matrix. The number of *B*-matrices is the same as the number of experimental effects. Since it is assumed that a connection between any two sources is of one type (forward, backward, or lateral), only one *B*-matrix per effect is necessary. The diagonal entries in the *B*-matrices allow modulation of the intrinsic (within source) connections. As described in [53], this makes it possible to model local changes in the excitability of a cortical area.

The “Review priors” button, also located in this part of the GUI, is for power users and opens another window making it possible to directly specify and refine priors on the neuronal model parameters and look at how they affect the model's dynamical responses. 

Several additional radio buttons located below the connectivity matrices are for toggling options specific to DCM-ERP.

- The “Dipolar symmetry constraints” option is useful for modelling bilateral symmetric sources (e.g., auditory cortices).
- “Optimise source locations” only works in combination with the “ECD” option and allows DCM more freedom with moving the dipoles as part of the optimisation process.
- “Lock trial-specific effects” ensures that all the changes in connectivity are the same. This is useful when there is a specific hypothesis that some experimental factor increases (or decreases) all connection strengths.

#### 6.3.4. Estimation

After model specification, the “Estimate” button can be pressed to invert the model. DCM then estimates the model parameters, which can take some time (typically from several minutes to an hour, depending on model complexity). One can follow the optimisation by observing the iterative model fit in a graphics window. In the MATLAB command window, the code will display the predicted and actual change in free energy (a bound approximation to the model's log-evidence that is being optimised) following each iteration. At convergence, DCM saves the results in a DCM file, by default named “DCM_ERP.mat”. The name can be changed by pressing “save” at the top of the GUI and saving to a different name.

#### 6.3.5. Results

**Figure 12:**
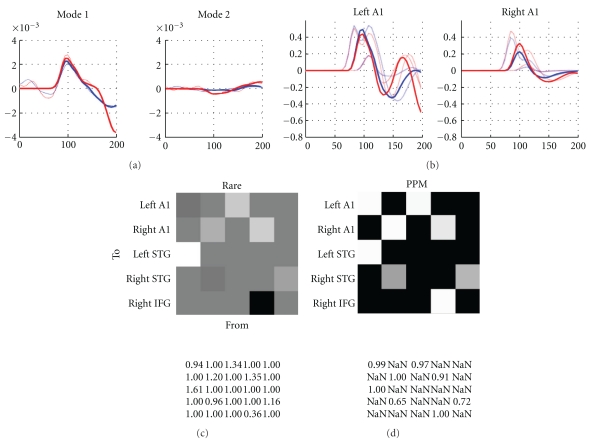
Examples from results display of DCM-ERP. The EEG data was taken from the mismatch negativity experiment available from the SPM website (a) “ERPs (mode)” display for the first two (out of eight) modes. The thin lines show the data and the thick lines the model prediction. Response to frequently presented tone is shown in blue and response to rare tone—in red. (b) “ERPs (sources)” display showing the predicted dynamics of two of the sources in the model (left and right auditory cortices). Each source consists of 3 neuronal populations. Response to frequently presented tone is shown in blue and response to rare tone—in red. (c) “Coupling (B)” display showing the posterior means for the gain modulations of the connections. (d) Posterior probability map from the same display showing for each modulated connection the probability that the effect of experimental conditions (frequent versus rare) on the connection was different from zero.

After estimation is finished, the results can be assessed by choosing from the pull-down menu at the bottom (middle). With “ERPs (mode)” one can plot, for each mode, the data for both evoked responses and the model fit (see Figure 12(a)). When selecting “ERPs (sources)”, the (posterior expectations of) dynamics in each source are plotted (see Figure 12(b)). The activities of the pyramidal cells (which are the reconstructed source activities) are plotted in solid lines, and the activities of the two other populations (inhibitory and excitatory interneurons) are plotted as dotted lines. The option “coupling (A)” will display a summary of the posterior distributions over the connections in the A-matrix. In the upper row (Figure 12(c)), the posterior means for all intrinsic connections are shown. As above, element **(i, j)** corresponds to a connection from area j to i. In the lower row (Figure 12(d)), for each connection, one can find the probability that its posterior mean is different from the prior mean, taking into account the posterior variance. With the option “coupling (B)” one can access the posterior means for the gain modulations of the connections. With “coupling (C)” one can see a summary of the posterior distribution for the strength of the input into the input-receiving source(s). On the left-hand side, DCM plots the posterior means for each area. On the right-hand side, the corresponding probabilities are provided. See Figure 12 for examples of these summaries based on the posterior (conditional) density over the model's hidden states and parameters. There are several other quantities that can be examined (see the SPM manual for further details).

There are two additional buttons to the right of the “Results” menu. The “Initialise” button makes it possible to assign parameter values as initial starting points for the inversion. These values are taken from another already estimated DCM, which the user can select. The “BMS” button opens the SPM batch tool for model selection. This tool allows one to perform Bayesian model comparison and Bayesian model averaging [9–11], which is usually the prelude to examining the parameter estimates of the selected model or Bayesian model average. 

This concludes our description of the specification and inversion of a DCM-ERP. We will now consider briefly the other DCMs and the interface features specific to these variants.

### 6.4. DCM for Steady State Responses

DCM-SSR is accessed by selecting “SSR” in the top-left drop-down menu. The second drop-down menu in the right of the top panel specifies (as with all DCMs) whether the analysis should be performed using a model that is linear in the states (ERP) or a conductance-based model (NMM) that is nonlinear in the states. The data selection and specification of between-trial effects are the same as for the case of ERPs, described above. The same is true for the electromagnetic model. DCM-SSR is commonly used with intracranial data, particularly from animal models and, therefore, the LFP option is especially relevant for this form of DCM. In the “Neuronal Model” the main difference from DCM-ERP is in the inputs. In DCM-SSR, inputs are not discrete events in time but endogenous noise sources. Therefore, the “onset” parameter is not relevant. The C matrix makes it possible to specify how the model sources are driven by noise. Usually, in this context, C is an identity matrix, prescribing endogenous fluctuations (noise) in all sources.

#### 6.4.1. Cross-Spectral Densities

In addition to variables discussed above, with DCM-SSR, it is necessary to select the frequencies that will be modelled. These could be part of a broad frequency range; for example, like the default 4–48 Hz, or one could enter a narrow band; for example, 8 to 12 Hz, which would model the alpha band. This specification is implemented via “frequency window (Hz)” boxes close to the bottom of the DCM window. After pressing the “invert DCM” button, the cross spectral densities are computed automatically (using the “spectral” toolbox in SPM [67]). The data features used for model inversion (i.e., those features generated or predicted by the model) include the auto-spectra and cross-spectra between channels (or modes). These data features are evaluated using a multivariate autoregressive model, which can accurately measure periodicities in the time-domain data. The resulting spectra are then presented as an upper-triangular, channel × channel matrix (or mode × mode), with autospectra on the main diagonal and cross-spectra in the off-diagonal terms.

#### 6.4.2. Output and Results

The “Results” menu provides several estimates. By examining the “spectral data”, one can see observed spectra in the matrix format described above. Selecting “Cross-spectral density” gives both observed and predicted responses. To examine the connectivity estimates one should select the “coupling (A)” results option, or for the modulatory parameters, the “coupling (B)” option. Also one can examine the input strength at each source by selecting the “coupling (C)” option, as in DCM-ERP. To examine the spectral input to these sources the “Input” option should be selected; this is a mixture of white and pink noise.

### 6.5. DCM for Induced Responses

DCM-IR models coupling within and between frequencies that are associated with linear and nonlinear mechanisms, respectively. DCM-IR is accessed by selecting “IND” in the top-left drop-down menu. Since this is a phenomenological DCM, the neural model menu is not relevant (i.e., it uses a simple bilinear approximation).

#### 6.5.1. Data Features

The data features (time-frequency response) modelled are formed from single-trial, epoched data. DCM-IR models the entire spectra, including both the evoked (phase locked to the stimulus) and induced (non-phase-locked) components. The “modes” variable has a different meaning in DCM-IR than in DCM-ERP. Here, these are not spatial modes but frequency modes that are taken from singular value decomposition of the time-frequency data concatenated across sources. The more modes selected, the more details of the time-frequency response will be modelled. However, when there are too many modes, DCM inversion is slow, and the higher order modes usually capture noise rather than physiologically meaningful dynamics. Usually the first 3-4 modes capture most of the interesting features so the default value of 8 should be more than sufficient in most cases.

#### 6.5.2. Electromagnetic Model

Unlike the DCMs above, DCM-IR does not model the data features in sensor space. The reason for this is that DCM-IR only models power and discards phase information. This makes it impossible to predict the sensor data given modelled source dynamics. Consequently, DCM-IR first inverts the electromagnetic model by using the pseudoinverse of the lead field matrix and then computes the source power, which is subsequently modelled. The IMG option is not relevant for this two-step procedure and, therefore, only the ECD and LFP options are available. When using the ECD option, the location parameters of the spatial model are not optimized. This means that DCM-IR will project the data into source space using the spatial locations specified by the user. We are currently considering more spatially specific methods for extracting source waveforms (e.g., beamforming). These methods will be implemented in the future.

#### 6.5.3. Neuronal Model

In DCM for induced responses, the A-matrices encode the strength of linear and nonlinear coupling between sources. The leftmost matrix in the first row specifies the linear connections. These are the connections where frequency energy in one source affects the dynamics of the same frequency in another source. Note that all connections in the model should be at least linear, so if a connection is present, the corresponding button in this matrix should be on. Also, the buttons on the leading diagonal of the matrix are always on because each node in the model has a linear intrinsic connection with negative sign. This ensures that induced activity has a tendency to dissipate. To the right of the linear connectivity matrix there is a nonlinear connectivity matrix. The idea here is the same. Note that the corresponding linear connection should be enabled as well. When a connection is nonlinear, a frequency in the source node can affect all the frequencies in the target node. Intrinsic connections can be made nonlinear as well so as to explain nonlinearities among putative subpopulations within each source.

The use of the input matrix and the onset parameters are similar to DCM-ERP. The B-matrices are also used as described above. It does not matter whether a connection is linear or nonlinear when specifying modulation by experimental effects. Hence, there is only one modulation matrix per experimental effect. Self-connections can be modified by experimental effects, thus the diagonal entries of the B-matrices can be toggled.

#### 6.5.4. Wavelet Transform

This button, located below the connectivity matrices allows one to transfer data into the time-frequency domain using a Morlet wavelet transform. One must also specify the frequency window that defines the desired frequency band and the number of cycles in the wavelet, which specifies the temporal-frequency resolution (see Appendix C.7). For the latter, we recommend values greater than 5 to obtain a stable estimation.

#### 6.5.5. Results

**Figure 13:**
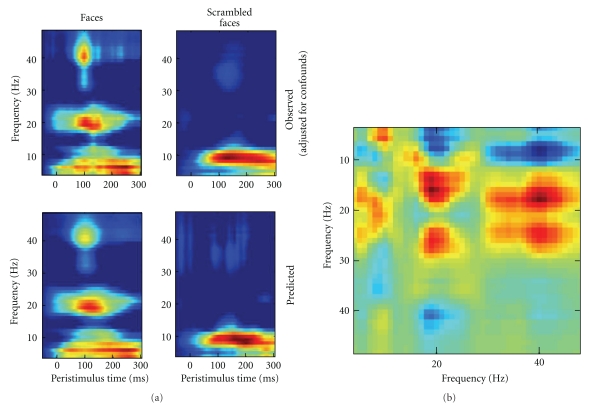
Examples from results display of DCM-IR. The MEG data was taken from the multimodal face perception experiment available from the SPM website. (a) Part of “Time-Frequency” display showing the observed and predicted time-frequency power for one of the model sources (right fusiform face area, rFFA). (b) Part of “Coupling (B-Hz)” display showing an example of a cross-frequency coupling matrix, in this case for connection between rFFA and right occipital source.

The “Frequency modes” option will display the frequency modes, identified using singular value decomposition of spectral dynamics in source space (over time and sources). “Time modes” will display the observed time courses of the frequency modes (dashed lines) and the model predictions (solid lines). Here, one can also see whether the activity picked up by the minor modes is noise, which is helpful for optimizing the number of modes. “Time-Frequency” will display the observed time-frequency power data for all prespecified sources (upper panel) and the fitted data (lower panel); see Figure 13(a) for an example. “Coupling (A-Hz)” will display the coupling matrices representing the coupling strength from source to target frequencies. These matrices are obtained by multiplying the between-mode coupling estimates with the frequency profiles of the modes [21]. “Coupling (B-Hz)” is similar to the above and reports modification of coupling by experimental effects (see Figure 13(b)). “Coupling (A-modes)” will display the coupling matrices between modes and the posterior probabilities that the coefficients are different from zero. This representation is useful for diagnostics when the inversion fails but the physiological interpretation is less straightforward. See the SPM manual for a more complete description and other options for reviewing the conditional estimates from DCM-IR.

A “Save as img” option allows one to save the cross-frequency coupling matrices as images. When analyzing a group of subjects one can use these images as summary statistics in SPM to find common features in coupling and coupling changes across subjects. The image names will include identifiers like “A12” or “B31” which relate to the source connection matrices; either the basic (A) or experimental effects (B).

### 6.6. DCM for Phase Coupling

**Figure 14:**
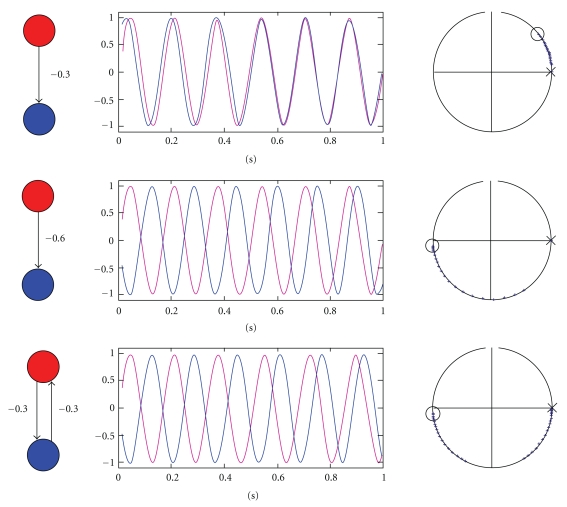
Examples for interactions between coupled oscillators that can be modelled with DCM-PHA. The left column shows the network structure used to generate the data in each row. The middle column shows the corresponding bivariate time series for two oscillators. The right column shows the corresponding phase diagrams on the unit circle with initial phases marked as a red cross for the first oscillator, and as a blue circle for the second. Subsequent phase evolutions are shown using dots. This figure was taken with permission from [23].

DCM-PHA is based on a weakly coupled oscillator model of neuronal interactions. This approach is used to describe dynamic phase changes in a network of oscillators; see Figure 14 for examples of this kind of dynamics. The influence that the phase of one oscillator has on the rate of change of phase of another is characterised in terms of a phase interaction function (PIF) as described in [23]. SPM supports PIFs specified using arbitrary order Fourier series. However, to simplify the interface, one is restricted to simple sinusoidal PIFs when using the GUI.

#### 6.6.1. Data Features

The data features (instantaneous phase) are computed from multiple trial, epoched data. Multiple trials are required so that the full state space of phase differences can be explored. This occurs because each trial is likely to contain different initial relative phase offsets. Information about different trial types is entered as it is with DCM-ERP. DCM for phase coupling is intended to model dynamic transitions toward synchronization states. As these transitions are short, it is advisable to model short time windows; the higher the frequency of the oscillations one is interested in, the shorter this time window should be. DCM for phase coupling will probably run into memory problems when using long time windows or large numbers of trials. Therefore, instead of a “modes” option (not relevant for DCM-PHA) there is a “subtrials” option which allows a subsampling of trials (i.e., using every second, every third, etc.).

#### 6.6.2. Electromagnetic and Neuronal Model

As with DCM-IR, DCM-PHA projects the data onto source space, using the spatial locations, provided by the user, and does not optimize the spatial model. ECD or LFP options are available. There is only one A-matrix (called “endog” meaning endogenous). If using the GUI, the phase interaction functions are given by *a*_*ij*_sin(*ϕ*_*i*_ − *ϕ*_*j*_), where *a*_*ij*_ are the connection weights that appear in the *A*-matrix and *ϕ*_*i*_ and *ϕ*_*j*_ are the phases in sources *i* and *j*. DCM for phase coupling can also be run from a MATLAB script. This provides greater flexibility, in that the phase interaction functions can be approximated using arbitrary order Fourier series. There is an example in the man/example_scripts subdirectory of the SPM distribution.

#### 6.6.3. Hilbert Transform and Results

Pressing the “Hilbert transform” button does two things. First, source data are bandpass filtered into the specified range. Second, a Hilbert transform is applied, from which time series of phase variables are obtained. The “Results” drop-down menu allows examining hidden states and parameter estimates; “Sin(Data)—Region I” plots the sine of the phase variable and the corresponding model fit for the *i*-th region. “Coupling (A)”and “Coupling (B)” will display the intrinsic and modulatory coupling matrices, respectively. The elements of A specify how quickly one source changes its phase to align with another. The corresponding entry in B shows how these values are changed by experimental manipulation.

## 7. Conclusion

In summary, we have reviewed the three main sorts of data analysis supported by the SPM software (and adopted in our scientific studies). These comprise analyses of sensor-level data to identify significant treatment effects in evoked or induced responses. The idea here is to use standard SPM (topological inference) to find significant regions in time × frequency or time × channels search spaces, whose *P*-values are properly adjusted for multiple comparisons and profound correlations over the implicit search spaces. These analyses can be the endpoint or a device to identify peristimulus time or frequency windows for subsequent source reconstruction or DCM. The second analysis domain we have looked at is source reconstruction, using either distributed or ECD forward models. These analyses allow one to identify where in the brain various treatment effects are expressed. Usually, distributed solutions are used simply to create summary statistic (time-frequency contrast) images for topological inference. Again, the ensuing SPM may be the final analysis or used to specify the number and location of sources that form the basis of DCM analysis. DCM and its variants have been reviewed as enabling hypothesis testing (model comparison) about the functional architectures subtending observed responses. There is an increasing portfolio of models available and DCM for electromagnetic signals is likely to be a focus of development for many years to come.

## Appendices

These appendices deal with the computer science and pragmatic aspects of handling data in SPM8. We include here substantial Appendices B and C on data formats and pre-processing, which are important parts of the analysis stream and share many protocols (and code) with other software platforms.

### A. SPM8 and FieldTrip

SPM developers have a formal collaboration with the developers of the FieldTrip toolbox (see Oostenveld et al., this issue) on many analysis themes. For example, SPM and FieldTrip share routines for converting data to MATLAB and some of the basic pre-processing and forward modelling for M/EEG source reconstruction. The SPM8 distribution contains a version of FieldTrip so that FieldTrip and SPM functions can be combined easily in custom scripts. For using FieldTrip functions within SPM, it is not necessary to install the full FieldTrip version. SPM and FieldTrip complement each other; SPM is geared toward very specific analysis tools whereas FieldTrip is a more generic repository of different methods that can be assembled in flexible ways to perform a variety of analyses. This flexibility of FieldTrip, however, comes at the expense of accessibility to a nonexpert user. FieldTrip does not have a GUI and its functions are accessed through custom scripts. By combining SPM8 and FieldTrip, the flexibility of FieldTrip can be complemented by SPM's GUI tools and batching system. Within this framework, power users can easily and rapidly develop specialized analysis tools with GUIs that can then also be used by nonproficient MATLAB users.

### B. Data Formats and Handling

In this appendix, our goal is to provide a comprehensive description of how the software can be used to pre-process M/EEG data, up to the point where one would use source reconstruction, DCM or statistical analysis of M/EEG channel data, as described in the main text.

Pre-processing functions can be called via GUI, the batch system or scripts. For scripting and command-line specification, we adhere to the principle of providing only one input argument to each function. This input argument is usually a MATLAB structure (struct) that contains all input arguments as fields. This approach has the advantage that the input does not need to follow a specific input argument order. If an obligatory input argument is missing, the function will invoke the GUI and ask the user for the missing argument. When using the GUI, a function is called without any input argument; that is, SPM will ask for the requisite input arguments.

#### B.1. SPM M/EEG Data Format

The first step of any analysis is the conversion of data from its native machine-dependent format to a MATLAB-based, common SPM format. A dataset in this format consists of two files: DAT-file which is a binary file containing just the data and MAT-file containing a structure with all the additional information related to the dataset.

There are two important programming devices that SPM uses when working with its M/EEG dataset: using object-oriented programming to access the header information and using memory mapping to access the data. The header data is stored in the MAT-file as a struct but when loaded by an SPM function into MATLAB memory, it is converted to an @meeg object. The internal structure of the header is thereby hidden from the user and is only accessed via special functions called “methods”. This has several advantages. First, using an object enforces internal consistency checks when the object is created or modified. If, for any reason, the header data becomes inconsistent, SPM will report this as soon the user tries to load these data. SPM will also report where the check flagged an inconsistency. If there is enough information available to fix the inconsistency, it is fixed on the fly. Second, using methods simplifies internal book-keeping, which makes it much easier to program functions operating on the M/EEG object. There is no need to check in higher level functions whether a particular header field is present and valid, as the object takes care of that. Third, using objects lends more flexibility to SPM developers because some details of the format can be changed without the need to update higher level SPM functions. It is therefore recommended that users writing scripts familiarise themselves briefly with the object functionality and work with the object rather than with the header struct directly.

The second important programming device is the use of memory mapping to access the data stored in the DAT-file. Memory mapping treats the data stored on the hard drive as if they were in memory, without the need to actually load all of the data at the same time. The technical details are handled by the operating system, but the important thing is that SPM can work with large files in a memory-efficient fashion. This means that SPM can handle large data sets without running into memory errors. The price can be a slight slowing in performance for some applications. Users writing custom code that needs to access the data are advised to do so in chunks (i.e., write a loop loading the data trial-by-trial or channel-by-channel in a continuous file) to take full advantage of memory mapping. Due to the way memory mapping is presently implemented, SPM only supports fixed-length trials (unlike for instance FieldTrip).

#### B.2. Conversion of Data

There are two ways to convert data from another format and create an SPM M/EEG dataset. The first is geared towards EEG and MEG datasets stored in their native formats or in formats of other analysis packages (e.g., EEGLAB). This conversion facility is based on “fileio” module (see http://fieldtrip.fcdonders.nl/development/fileio), which is shared between SPM8, FieldTrip and EEGLAB toolboxes and jointly developed by the users of these toolboxes. At the moment, most common EEG and MEG data formats are supported. For some formats, it might be necessary to install additional MATLAB toolboxes (there is an error message if these toolboxes are missing). If the data format is not recognized by “fileio”, it will attempt to read the file using the Biosig toolbox (http://biosig.sourceforge.net/), if available. This can work for some EEG systems, particularly clinical ones. One of the consequences of using Biosig as a fallback option is that error messages from Biosig or mentioning Biosig can appear if the data format is not supported. If necessary the “fileio” toolbox can be extended easily to support additional formats.

The majority of formats supported by “fileio” are also automatically recognized. Therefore, format-specific details are hidden in the library and SPM can deal with conversion in a generic fashion. After selecting a file for conversion, the user can let SPM and fileio convert it automatically and read all the data in the file. There is also a facility to configure parameters for conversion. This is useful for instance when only a part of a large data file needs to be read or only a subset of channels are of interest.

In many cases, it is necessary to work with data that is not stored in any standard format; for instance, data stored as a MATLAB variable or an ASCII file. There are two options for importing these data, depending on whether the user can write a MATLAB script or wants to use the GUI. For users who would like to rely on the GUI, we suggest they assemble their dataset in EEGLAB (Delorme et al., this issue) and then save it and convert to SPM as with any other format. The developers of EEGLAB have invested a lot of effort into making it possible to build a dataset from scratch, without using the command line or scripts.

For those who prefer to work with MATLAB scripts, the most straightforward way to convert custom data is to create a simple FieldTrip raw data structure and then use SPM's spm_eeg_ft2spm.m function to convert this structure to SPM. Missing information can then be supplemented using @meeg methods and SPM functions.

FieldTrip raw struct must contain the following fields.

- .fsample—sampling rate (Hz).
- .trial—cell array of trials containing matrices with identical dimensions (channels × time).
- .time—cell array of time vectors (in sec)—one cell per trial, containing a time vector the same length as the second dimension of the data. For SPM, the time vectors must be identical.
- .label—cell array of strings; a list of channel labels. Same length as the first dimension of the data.

#### B.3. Data Reviewing and Augmentation

Following conversion and throughout pre-processing, the data can be reviewed using the SPM8 reviewing tool. This tool allows the user to visualise data and source reconstructions and review or modify much of the header information on the dataset (e.g., channel labels and types). When reviewing continuous data, it is possible to add events manually (e.g., mark epileptic spikes). It is also possible to mark trials and channels as bad.

fMRI artefact rejection and sleep scoring toolbox (FASST, http://www.montefiore.ulg.ac.be/~phillips/FASST.html) can be used as an alternative to the SPM8 reviewing tool to speed up reviewing of long continuous multichannel datasets (see Leclercq et al., in this issue).

SPM tries to do its best to extract information automatically from the various data formats. In some cases it can also supplement the converted dataset with information not directly present in the raw data. For instance, SPM can recognise common EEG setups (extended 10–20, Biosemi, EGI) based on channel labels and assigns “EEG” channel type and default electrode locations for these cases. However, there are data types that are either not yet supported in this way or do not contain sufficient information for SPM to make automatic choices. Furthermore, channel labels do not always correctly describe the actual electrode locations in an experiment. In these cases, further information needs to be supplied by the user. The SPM “Prepare” tool, accessible from the reviewing tool, makes it possible to augment and modify the data in several important ways. First, it is possible to review and modify channel types. Channel types are important in SPM because they determine how SPM interprets the information in the channels and the dataset as a whole. For instance, if EEG or MEG channels are present in the dataset, SPM will expect sensor locations to be defined when this dataset is loaded for 3D source reconstruction or DCM analysis. Second, using the “Prepare” interface one can load individual EEG electrode locations and individual head shapes used for MEG coregistration. Sensor positions and fiducials for MEG are extracted from the raw data automatically and are already present after conversion. However, sometimes head shape measurement outside the scanner is used to relate the locations of MEG head position indicator coils to anatomical fiducials that can be marked on an MRI. SPM handles this by allowing the user to load the head shape via “Prepare”. Third, an important function of the “Prepare” interface is to create 2D channel layouts used for creating topographical scalp plots and converting M/EEG data to images for statistical analysis. These layouts can be created automatically by projecting 3D sensor locations to 2D, but they can also be built manually by the user or loaded from a fixed template. When possible, SPM creates 2D layouts automatically at conversion. Without meaningful 2D coordinates, all SPM functions will work correctly but the channels will be laid out in a topographically meaningless rectangular pattern.

### C. Data Processing

In this appendix, we will describe the high-level SPM functions which are used for pre-processing of converted M/EEG data (e.g., epoching, filtering, averaging). The majority of these functions have functionality similar to that implemented in commercial and other open source M/EEG analysis packages. Here, our aim was to make SPM self-contained so that the users will not usually need other software to prepare their data for SPM analyses proper. Where possible, SPM shares low-level code with FieldTrip. The general syntax is the same for all functions. If called from the command line, and if no input arguments are specified, the function will behave exactly as if called from the GUI. However, on the command line (or from a script) it is possible to supply input arguments. When all required input arguments are specified, the function will run without user interaction. In this way, one can write a noninteractive script. Input arguments are provided in a struct called S, whose fields contain the arguments. One of the fields (usually S.D) specifies the input dataset and can be either an @meeg object or file name. The majority of SPM pre-processing functions create a new output dataset, rather than modify the input dataset. This is somewhat wasteful in terms of disk space, but makes it possible to backtrack and try different options for every analysis step without the need to recompute. The filenames of the output MAT- and DAT-files are generated by prepending a single letter to the input file name. In the example of epoching, this would be an “e”. The idea is that after calling a sequence of functions on a file, the file name encodes the pre-processing steps that were called to produce this file. Note that another way of calling SPM functions and specifying all input parameters is to use the batch interface.

The order of the pre-processing steps is not fixed and depends on different considerations. For instance, it is important to set the channel types correctly prior to filtering because channels with undefined (“Other”) type are not filtered. The logic here is to preserve information in channels for which filtering is not relevant (e.g., trigger channels).

#### C.1. Epoching

Epoching cuts out little chunks of continuous data and saves them as “single trials”. In M/EEG research, this is a standard data selection procedure to remove long gaps between trials. An epoch starts at some user-specified pre-stimulus time and ends at some poststimulus time; for example, from −100 to 400 milliseconds in peristimulus time. By default, the epoched data will also be baseline corrected; that is, the mean of the pre-stimulus time is subtracted from the whole trial.

The epoching function can implement two ways of specifying trials to epoch. The first is to specify trials using labelled events stored in the file. The user should define the prestimulus and poststimulus interval, and specify the events (triggers) around which the epochs will be “cut”. SPM identifies events by their “event type” and “event value”. These are strings or numbers, which the software used by the EEG or MEG vendor produces when generating the measurement file. These can sometimes look strange but are usually the same for a given system, so it is only necessary for the user to find out once which triggers are relevant for the experiment. It is also necessary to define a “condition label” for each trial type, which can be any string. This is the label that SPM will use to indicate the trial type of a trial at later processing stages. It is possible to use several types of triggers for defining trials with the same label.

For most users, the epoching described above is the most convenient but there are situations when a single trigger is not sufficient to specify the trial type. This happens, for instance, when the epoching is done around the stimulus but the trial type also takes into account the response that comes later. This situation can be dealt with by epoching around the stimulus trigger and defining a single trial type but relabeling the trials at a later stage. Relabeling can be done using @meeg object methods or the reviewing tool. In even more complicated cases, or when there is no event information available in the raw data, it is up to the user to write a custom script for trial definition and specify explicitly where each trial is located in the measured time series.

This script should generate an *N* × 2 so-called “trl” matrix, where each row contains the start and end of a trial (in samples). Optionally, there can be a third column containing the offset of the trigger, with respect to the trial. An offset of zero (the default) means that the first sample of the trial corresponds to the trigger. A positive offset indicates that the first sample is later than the trigger and a negative offset indicates that the trial begins before the trigger. In SPM, the offset should be the same for all trials. The need to specify a whole column is for interoperability with FieldTrip, where trials can have different time axes. In addition, condition labels need to be specified either as a single string or a cell array of strings with a label per trial.

#### C.2. Filtering

Continuous or epoched data can be filtered, over time, with a lowpass, highpass, bandstop, or bandpass-filter.

#### C.3. Downsampling

The data can be downsampled to any sampling rate.

#### C.4. Baseline Correction

This function subtracts the baseline from channel data. The baseline period needs to be specified in ms; for example, [−100 0].

#### C.5. Artefact Detection and Rejection

Some trials not only contain (neuronal) signals of interest, but also large signals from other sources, like eye movements or muscular activity. These signal components are referred to as artefacts. There are many kinds of artefacts and methods for detecting them. The artefact detection function in SPM is, therefore, extendable and can automatically detect and use plugin functions that implement particular detection schemes. Simple algorithms presently implemented include thresholding of the data, thresholding of the difference between adjacent samples (to detect jumps), thresholding peak-to-peak amplitude, and detection of flat segments. Channels containing artefacts in a large proportion of trials are automatically marked as bad. Note that the function only indicates which trials are artefactual or clean and subsequent processing steps (e.g., averaging) will take this information into account. However, no data are actually removed from the DAT-file.

#### C.6. Montage Application

The montage function in SPM basically multiplies the channel data by a matrix. This can be used to implement various processing steps. One common example is EEG re-referencing: Presently, for source analysis and DCM, EEG data should be re-referenced to the channel average, to meet the assumptions of the forward model used. For sensor level analysis, it is sometimes useful to use a reference that emphasizes the effects of interest. Other common uses are deriving bipolar channels, when data are recorded with common reference (e.g., EMG, EOG), and adding or removing channels.

The montage function can also be used for denoising and artefact reduction. Removing artefactual components, using projections derived from independent component analysis (ICA), signal space projection (SSP), or the application of synthetic gradients implemented in the CTF MEG system, are all particular cases of montage. An important point here is that the application of the montage function often changes the dimensionality of the data or the relationship between sensors and channels. This should be taken into account when computing the forward model for source analysis. Presently, this is only done for MEG, where the montage function automatically updates the sensor representation to ensure consistency with the data. Similar mechanisms for EEG will be implemented in the future but for now, removing too many spatial components from the data should be avoided, especially with a small number of channels.

#### C.7. Time-Frequency Analysis

Time-frequency analysis makes it possible to study oscillatory neural activity that appears consistently at particular times, relative to the event of interest; even if this activity is not phase locked to the event and therefore averages out in conventional analyses of evoked responses. This usually involves transforming the data to the frequency domain in short (possibly overlapping) time windows. Combining the spectra for all time windows yields 2D images that can be analysed using SPM statistical machinery (topological inference). All methods of time-frequency analysis are inherently limited by the uncertainty principle; stating that the resolution in time is inversely related to frequency resolution. Therefore, in order to estimate the frequency of particular response more precisely, one should examine longer data segments and sacrifice time resolution. The opposite holds when trying to localize a signal precisely in time. Different methods of time-frequency analysis handle this resolution trade-off slightly differently and are, therefore, optimal for certain kinds of signals and suboptimal for others. For example, in the 300 ms following an event (which is more or less the reaction time in simple tasks) there are only three cycles at alpha frequency (10 Hz) and 6 cycles at beta frequency (20 Hz). Thus, analysis of event-related activity at these frequencies requires a method that can reliably estimate power and phase of an oscillation, based on a small number of cycles. Wavelet analysis using Morlet wavelets compares the signal of interest with short segments of an oscillation multiplied by a Gaussian. This results in very high time resolution and makes wavelet analysis the method of choice for low frequencies. However, when we look at higher frequencies, especially in high gamma range (above 50 Hz), the number of cycles is no longer an issue. In 300 ms, there are 24 cycles of an 80 Hz oscillation. The main problem is now the fact that high gamma activity may be highly variable in timing and precise frequency. Therefore, the high time and frequency resolution of wavelets is rather a disadvantage in this case and results in patchy time-frequency plots with characteristic “fingers”. Multitaper spectral analysis is the optimal way to “smooth” the spectral estimates in both time and frequency, and thereby gain more power for detecting high gamma activity. This method is based on premultiplying the data with a series of tapers optimised for producing uncorrelated estimates of the spectrum in the given frequency band. This makes it possible to sacrifice some of the frequency resolution in a well-controlled manner to gain a higher signal-to-noise ratio, by effectively multiplying the number of trials by the number of tapers used.

The above example shows why no single method of estimating power over time and frequency is optimal for all applications and even within a method parameters need to be optimized to get the best spectral estimate for a particular dataset. SPM makes it possible to perform such optimization by offering a flexible and extendible interface based on the matlabbatch GUI. Different spectral estimation methods are implemented as automatically detectable plugins. Algorithms presently implemented include continuous Morlet wavelet transform, Hilbert transform, and tapered fast Fourier transform (FFT), including multitaper spectral estimation. The result is written to one or two result files, one containing the instantaneous power and the other (optional) containing phase estimates (phase estimation is not possible for some algorithms). One can select the channels and frequencies for which power and phase should be estimated. In the future, additional algorithms will be added and we are planning to share much of the low-level code with FieldTrip.

#### C.8. Averaging

Averaging of single trial data is the crucial step to obtain the evoked response. When averaging single trial data, single trials are averaged within trial type. Power and phase data of single trials can also be averaged by using the SPM averaging function.

A special feature implemented in SPM is robust averaging. This is a rather simple case of robust general linear modelling [68]. The idea is that for each channel and time bin (or time-frequency pixel), the distribution of values over trials is used to downweight outliers, when computing the average. This suppresses artefacts restricted to narrow time and frequency ranges, without rejecting whole trials. Moreover, a clean average can be computed with no clean trials; given that the artefacts do not consistently overlap and only corrupt (different) parts of trials.

The robust averaging algorithm estimates weights, lying between 0 and 1, that indicate how artefactual a particular sample in a trial is. Later, when averaging to produce evoked responses, each sample is weighted by this number. If the weight of a sample is close to zero, it has little influence on the average. The sensitivity of the algorithm to outliers is controlled by the “offset for the weighting function” parameter. This value (3 by default) defines the weighting function used for averaging the data. This will preserve roughly 95% of data points drawn randomly from a Gaussian distribution. Another choice the user should make is whether to compute the weights by condition (as opposed to all the trials). If one condition has fewer trials than the others, it is generally safer to estimate the weights separately for each condition; otherwise, evoked responses in the rarer condition will be downweighted and become more similar to the more common condition(s). The weights can be saved as a separate dataset, which is useful for finding out what parts of the data were down-weighted and adjusting the parameters if necessary. Robust averaging can be applied to either time or time-frequency data. In the case of time data, if a low-pass filter was applied before averaging, one should apply it again (after averaging) because the differential weighting of adjacent points may introduce high frequencies.

### D. Additional Utility Functions

#### D.1. Grand Mean

The grand mean is usually understood as the average of evoked responses over subjects. The grand mean function in SPM is typically used to compute this, but can also be used to average over multiple EEG files; for example, multiple sessions of a single subject. There is an option to weight by the number of trials in each file (suitable for averaging across sessions within a subject) or do unweighted averaging (suitable for averaging across subjects).

#### D.2. Merge

Merging several M/EEG files can be useful for concatenating multiple sessions of a single subject. Another use is to merge files and then use the reviewing tool to display data from different files in the same graph. The function has a mechanism for recoding condition labels so that, for instance, all trials coming from the same original file will have an identifier added to their condition label in the merged file.

#### D.3. Multimodal Fusion

SPM supports datasets containing simultaneously recorded MEG and EEG. For imaging source reconstruction, it is possible to use both modalities to inform the source solution. Usually, combined MEG/EEG data is contained within the same raw dataset and can be preprocessed together from the beginning. If this is not the case, the fusion functionality makes it possible to combine two datasets with different channels into a single dataset; given that the sets of channels do not overlap and the datasets are identical in the other dimensions (i.e., have the same sampling rate and time axis, the same number of trials, and the same condition labels in the same order). This function can be used to create a multimodal dataset from separately recorded MEG and EEG, for experiments with highly reproducible ERP/ERFs.

#### D.4. Rescaling and Baseline Correction of Time-Frequency

Usually, raw event-related power is not the most informative thing to look at (although contrasts of raw power between conditions can be informative). To see the event-related effects better, the power can be either transformed or baseline-corrected separately for each frequency. There are several different ways to do this. “LogR” (log-ratio) method first computes the log of power and then baseline-corrects and scales the result to produce values in dB. “Diff” just does simple baseline subtraction. “Rel” expresses the power in percentage of the baseline units. Finally, the “Log” and “Sqrt” options just compute the respective functions without baseline correction. If necessary, the baseline period needs to be specified. The baseline can also be taken from a different dataset. This allows a baseline condition rather than baseline period.

#### D.5. Contrast of Trials

As an extension to the averaging functionality, SPM can also be used to compute linear combinations of single trials or evoked responses. A simple example is computing the difference between two evoked responses with a contrast weight vector [−1 1]. Another example is pooling across conditions when several trial types are to be treated as one. In this case there is an option to weight the contrast coefficients by the number of replications in each trial type. In principle, any contrast that can be formulated in the GLM framework can be applied to the data. This can be useful for a “localisation of contrast” approach to source reconstruction.

#### D.6. Copy

This function makes it possible to make a copy of a dataset. Note that one cannot just copy and rename the files in the usual way because the name of the data file is stored in the header file and this should be updated. The user will be asked to specify the new dataset name.

#### D.7. Sort Conditions

In many cases in SPM, the order of the conditions in the file is important (e.g., in 3D source reconstruction and in DCM). This function makes it possible to change the specification of the order (without actually changing the data file). Subsequently, every time the order of the conditions is called, the order thereby specified will be used. For instance, if one sorts conditions in an epoched file and then averages it, the conditions in the average file will be ordered as specified. If trials were originally defined by selecting events from a list, then the order in which the selection was made will be preserved.

#### D.8. Remove Bad Trials

This function physically removes trials marked as bad from a dataset. This can be useful, for instance, before time-frequency computation, as processing bad trials entails an unnecessary overhead. Also, when it is necessary to remove trials from a dataset (e.g., to remove unused conditions), these trials can be first marked as bad and then removed using this function.

#### D.9. Crop

This function makes it possible to trim the time axis and/or frequency axis (if available). This is useful when computing time-frequency decompositions and padding the trials with extra data to prevent edge effects. The padding can then be removed using the crop functionality.

### E. Script Generation

Data pre-processing in SPM can be automated with MATLAB scripts. Furthermore, these scripts can be generated automatically. To do this, one dataset needs to be analyzed first, using the GUI or batch system. Whenever a pre-processing function is called, all the input arguments, once they have been assembled by the GUI, are stored in the dataset's “history”. This history can then be accessed via the reviewing tool to not only see which functions have operated on a dataset, but also to generate a script that reproduces the same operations. The big difference is that, this time, no more GUI interactions are necessary because the script already has all the input arguments, which were provided during the first run. Since the script is automatically generated, it might not be as concise and elegant as a script written by a programmer. For instance, automatically generated scripts sometimes include specification of large montage matrices or long lists of channel labels. But with a basic understanding of MATLAB programming, such scripts can be shortened and simplified.

Automatically generated scripts can be used not only to repeat an analysis, but can also be treated as a template for other analyses. For instance, file names appearing in the script can be changed to preprocess a different subject or (with some adjustments) automatically generated code can be placed in a loop to cycle over a large number of subjects. Modifying automatically generated scripts is an easy way to get started with programming in SPM for M/EEG.

### F. Example Datasets

We provide several example datasets on which SPM methods can be tested. The full list of datasets with links can be found under http://www.fil.ion.ucl.ac.uk/spm/data/. Detailed instructions for analysing these datasets are available in the SPM manual (http://www.fil.ion.ucl.ac.uk/spm/doc/spm8_manual.pdf).

### G. Toolboxes and Contributing to SPM

We try to maintain SPM for M/EEG as generic, well-structured, and fully documented code. This is important to ensure stability and facilitate future development. However, this can make it more difficult for users to contribute their own tools to SPM. Therefore, SPM includes a protocol for the users to add their own code as toolboxes. These toolboxes are detected automatically and added to the “Toolbox” menu in the main SPM window. The complete list of toolboxes can be found at http://www.fil.ion.ucl.ac.uk/spm/ext/.

Presently, there are two toolboxes for SPM for M/EEG included in the SPM distribution.

#### G.1. MEEGtools Toolbox

This toolbox includes some useful functions contributed by SPM developers and power users. Many of these functions combine SPM and FieldTrip functionality. Other functions solve system-specific problems that cannot be handled by the main SPM code. For example, there is a set of functions for topography-based artefact correction that have been found to work well for eye blinks [69] and transcranial magnetic stimulation artefact in the EEG [70].

#### G.2. Beamforming Toolbox

Functions in this toolbox make it possible to perform source reconstruction using beamforming methods in the time [71] and frequency [72] domains and extract source activity using beamformer spatial filters. They make use of SPM-generated forward models (see “Source reconstruction”) and (where relevant) generate images that can be entered into the SPM statistics pipeline. Some of these functions are based on FieldTrip code and others are being developed by one of the authors (G. Barnes). In the future, this code is likely to be reorganised with some of the functionality transferred to SPM 3D source reconstruction interface and some integrated into FieldTrip.

We welcome code contributions from SPM users. Single general-use functions can be contributed to MEEGtools. Users who develop useful sets of functions may consider maintaining and distributing them as an external SPM toolbox. Active external developers, who are sufficiently familiar with the code, can be granted write access to our version control system and contribute to development and improvement of the code.
